# Design, Synthesis, and Evaluation of Novel Thiazole-Based Peptidomimetic Compounds as Potent SARS-CoV‑2 Main Protease Covalent Inhibitors

**DOI:** 10.1021/acsinfecdis.6c00019

**Published:** 2026-05-20

**Authors:** Weile Yin, Wai-Po Kong, Siu-Lun Leung, Zhiguang Liang, Yu Wai Chen, Kwok-Yin Wong

**Affiliations:** Department of Applied Biology and Chemical Technology, 26680 The Hong Kong Polytechnic University, Hung Hom, Kowloon, Hong Kong, China

**Keywords:** peptidomimetic inhibitors, covalent inhibitors, thiazole-based compounds, SARS-CoV-2 M^pro^, structure−activity relationship

## Abstract

The main protease (M^pro^) of SARS-CoV-2, essential for the replication of the virus, is a critical target for antiviral drug development. Herein, we developed a series of thiazole-based peptidomimetic compounds to identify potent and selective inhibitors targeting M^pro^. Compound AD06 (IC_50_ = 163.3 ± 43.5 nM) exhibited the most potent inhibitory potency against SARS-CoV-2 M^pro^, which is comparable to Nirmatrelvir (IC_50_ = 160.2 ± 15.1 nM) and the lead compound MC12 (167.4 ± 28.6 nM). Crystallographic analysis revealed that AD06 and AD05 have favorable binding conformation with interactions at S1, S2, and S3/4 subsites. Notably, AD05 (EC_50_ = 3.22 ± 0.61 μM) displayed stronger antiviral activity than AD06 (EC_50_ = 25.58 ± 0.71 μM), despite its weaker enzymatic inhibitory activity (IC_50_ = 306.5 ± 44.2 nM). Neither of them (CC_50_ > 100 μM) showed notable cytotoxicity in Vero E6 cells, highlighting AD05 as a promising candidate for further development against SARS-CoV-2.

## Graphical abstract

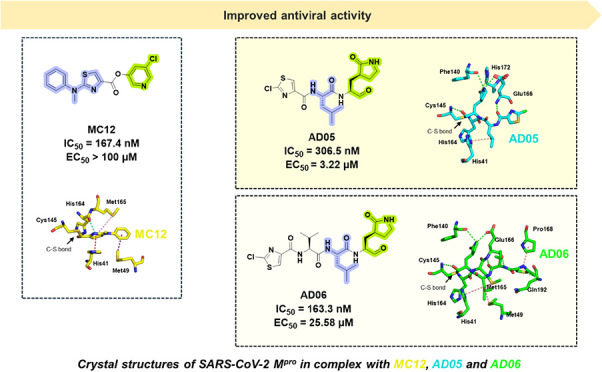

Developing specific antiviral therapies that target the key stages of the SARS-CoV-2 replication cycle has been proven to be effective in curbing viral replication and propagation within the host, thereby limiting its spread.^1–4^ The M^pro^ (main protease, alternatively termed 3CL^pro^) has become a major focus of research owing to its indispensable role in the process of viral replication.^5–7^ As one of the dimeric cysteine proteases, the active pocket of SARS-CoV-2 M^pro^ is situated in the gap between domain I and domain II of each monomer unit.^6,8–11^ During viral replication, M^pro^ cleaves 11 conserved sites of the polyprotein chains 1a and 1ab, such as the junction between nonstructural protein 5 (nsp5) and nsp16.^12–14^ There are multiple indispensable nonstructural proteins, such as RNA-dependent RNA polymerase (RdRP, nsp12) and viral helicase (nsp13),^12–14^ that are produced through this cleavage process. This makes M^pro^ important for the early replication process and a potential inhibition target to disrupt the entire downstream production of viral components.

Various approaches have been adopted to discover the potent inhibitors of SARS-CoV M^pro^ and SARS-CoV-2 M^pro^, such as rational drug design,^15^ drug repurposing,^16^ mass spectrometry,^17,18^ and high-throughput screening (HTS).^19^ Recent years have witnessed significant progress in the research of SARS-CoV-2 M^pro^ inhibitors, which have been comprehensively reviewed in several articles.^20,21^ These inhibitors can be classified into two major structural categories: peptidomimetic derivatives represented by nirmatrelvir that mimic native viral substrates, and nonpeptidic small-molecule compounds such as masitinib, MAC-5576, and MC12 (Figure 1). The nonpeptidic small molecules masitinib^16,22^ and MC12 ^23^ contain a thiazole moiety and possess multiple biological properties, including antitumor^22^ and antiviral^16,23^ activities. Structural analysis of the reported SARS-CoV-2 M^pro^ inhibitors yields the following insights: Peptidomimetic inhibitors that are developed via a structure-based strategy usually form covalent bonds with the key catalytic residue Cys145 in the active site to block the enzymatic mechanism. On the other hand, small-molecule inhibitors acquired from high-throughput screening (HTS) and drug repurposing strategies are further categorized into covalent and noncovalent compounds. Covalent inhibitors have electrophilic functional warheads to establish stable covalent linkages with the Cys145 residue within the active cavity.^24,25^ By comparison, noncovalent inhibitors primarily interact with the S1 subsite, which is a pivotal region responsible for specific recognition and binding at the catalytic domain. Furthermore, such noncovalent lead compounds can be chemically transformed into covalent inhibitors via the introduction of tailored reactive functional groups.^26^

**Figure 1:**
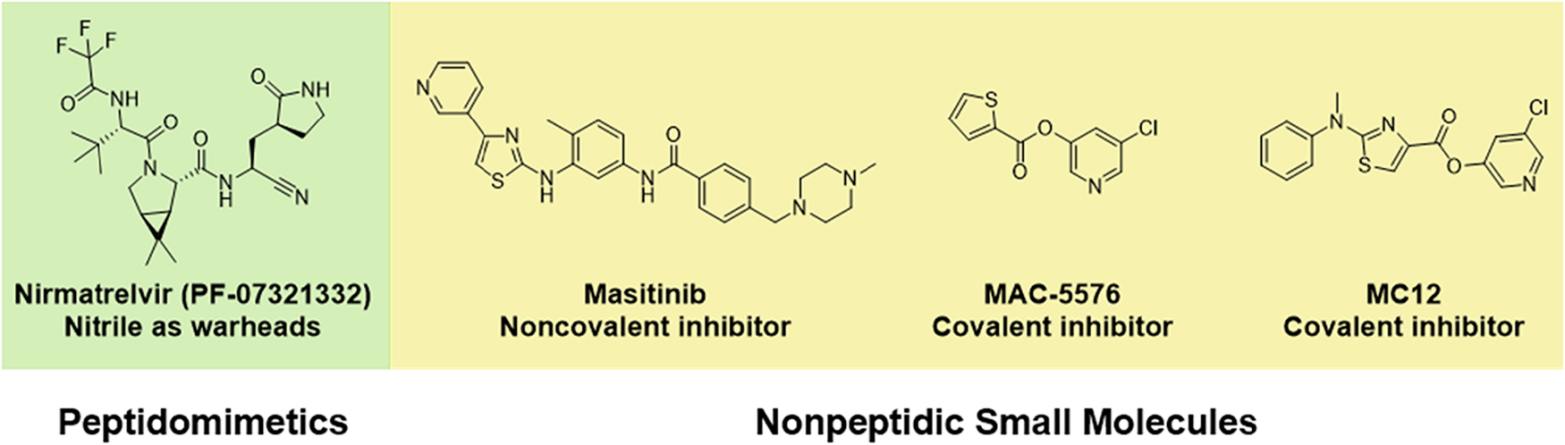
Representative structures of SARS-CoV-2 M^pro^ inhibitors.

Despite the extensive progress made in exploring various SARS-CoV-2 M^pro^ inhibitors, several limitations of these compounds remain. For instance, peptidomimetic inhibitors have been documented to possess relatively short plasma half-lives *in vivo*.^24^ Though clinically authorized SARS-CoV-2 M^pro^ inhibitors like nirmatrelvir^27^ demonstrate robust anticoronavirus efficacy, the need to coadminister nirmatrelvir with ritonavir^28^ (one of the CYP3A inhibitors) restricts their application in certain patients (*e.g*., those with hepatitis^29^). Accordingly, the discovery and design of new M^pro^ inhibitors to overcome the above bottlenecks are urgently needed, which also provide great prospects for the innovation of antiviral treatment regimens. In this study, we developed a novel series of thiazole-containing peptidomimetic inhibitors targeting SARS-CoV-2 M^pro^ by modifying our previously reported nonpeptidic small-molecule compound MC12 (Figure 1),^30^ and the designed compounds emerge as a promising scaffold for future drug development.

## Results and Discussion

### Design, Synthesis, and Optimization of Peptidomimetic SARS-CoV-2 M^pro^ Inhibitor with Thiazole Moiety

Structural study revealed that the SARS-CoV-2 M^pro^ inhibitor Masitinib (PDB ID: 7JU7) and MAC-5576 (PDB ID: 7JT0) have a similar binding mechanism. The *N*-phenylthiazol-2-amine fragment of Masitinib is anchored within an identical subsite as the thiophene moiety of MAC-5576 in the SARS-CoV-2 M^pro^ binding pocket.^18,31^ Molecular docking studies further support similar binding of the two molecules to the M^pro^ pocket—the pyridine nitrogen of MAC-5576 forms a hydrogen-bonding network with the imidazole group of His163, which is similar to the interaction of the pyridine-3-yl ring of Masitinib with the S1 subsite.^18,31^ To leverage these insights, we previously employed a scaffold fusion strategy to design compound MC12 (Figure 2A).^30^ The inhibitory mechanism of MC12 was elucidated through X-ray crystallography of the SARS-CoV-2 M^pro^-MC12 complex (PDB ID 9M2V, Figure 2B).^30^ Through nucleophilic attack, MC12 covalently bind to the residue Cys145. Additionally, the thiazole ring of MC12 stabilizes the complex through π-π stacking with both His164 and His41, while the nitrogen atom on the thiazole group participates in hydrogen bonding with the backbone of His164. Collectively, MC12 ^30^ exhibits a covalent inhibitory mechanism closely consistent with that of MAC-5576,^32^ as reported in the literature.^30^

**Figure 2:**
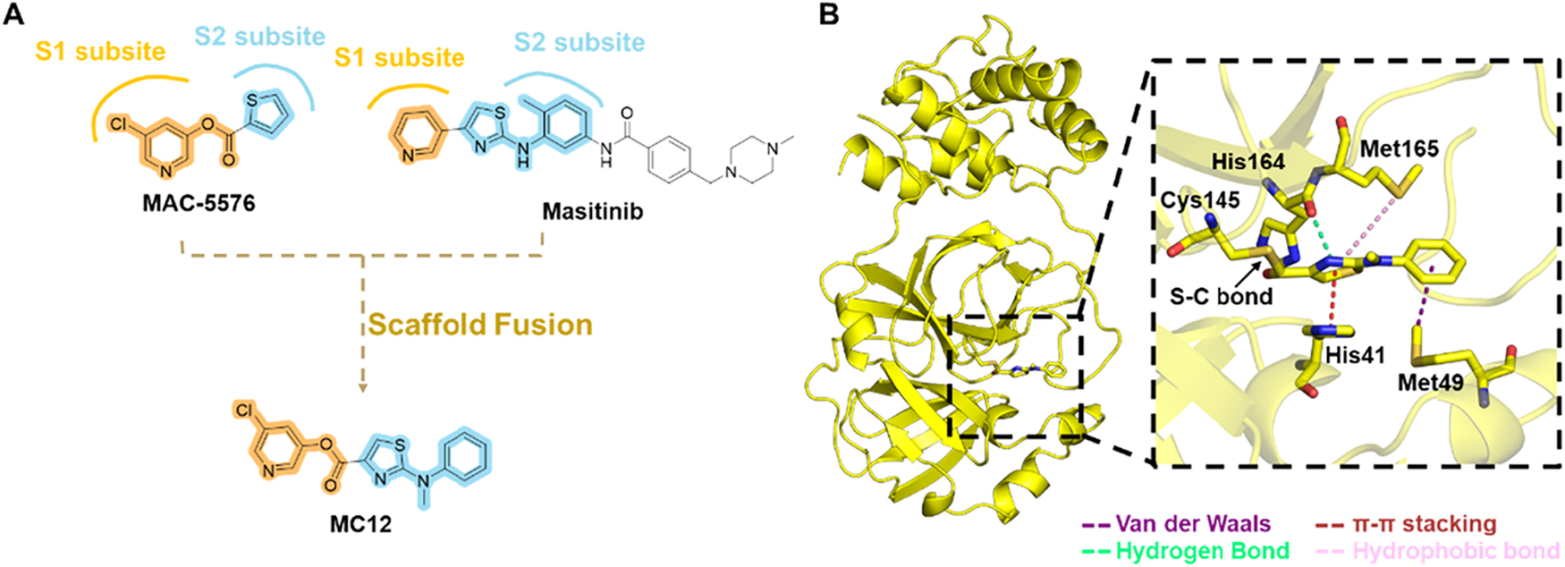
(A) Design strategy of the thiazole-based compound MC12. (B) X-ray crystal structure of MC12 in complex with SARS-CoV-2 M^pro^ (PDB ID: 9M2 V).

To overcome the weak antiviral activity, we systematically modified the structure of MC12. The crystal structure of MC12 in complex with SARS-CoV-2 M^pro^ revealed that nucleophilic attack by Cys145 on the carbonyl group of MC12 leads to the formation of a C-S covalent bond, which subsequently undergoes elimination of 3-chloro-5-hydroxypyridine as the leaving group. This suggests that once the 3-chloro-5-hydroxypyridine fragment of MC12 is released from the binding pocket, the remaining 2-(methyl­(phenyl)­amino)­thiazole moiety might diminish the binding affinity and reduce the recognition of the S1 subsite. To address this issue, we focused on structural optimization by modifying the pyridinyl ester warhead of MC12 to an aldehyde block (Figure 3). The resulting compound, AD01, incorporates a new moiety designed to mimic glutamine, thereby enhancing binding specificity to the S1 subsite. On top of that, we also altered the warhead with different functional groups to explore the impact on the specificity and binding affinity for M^pro^.

**Figure 3:**
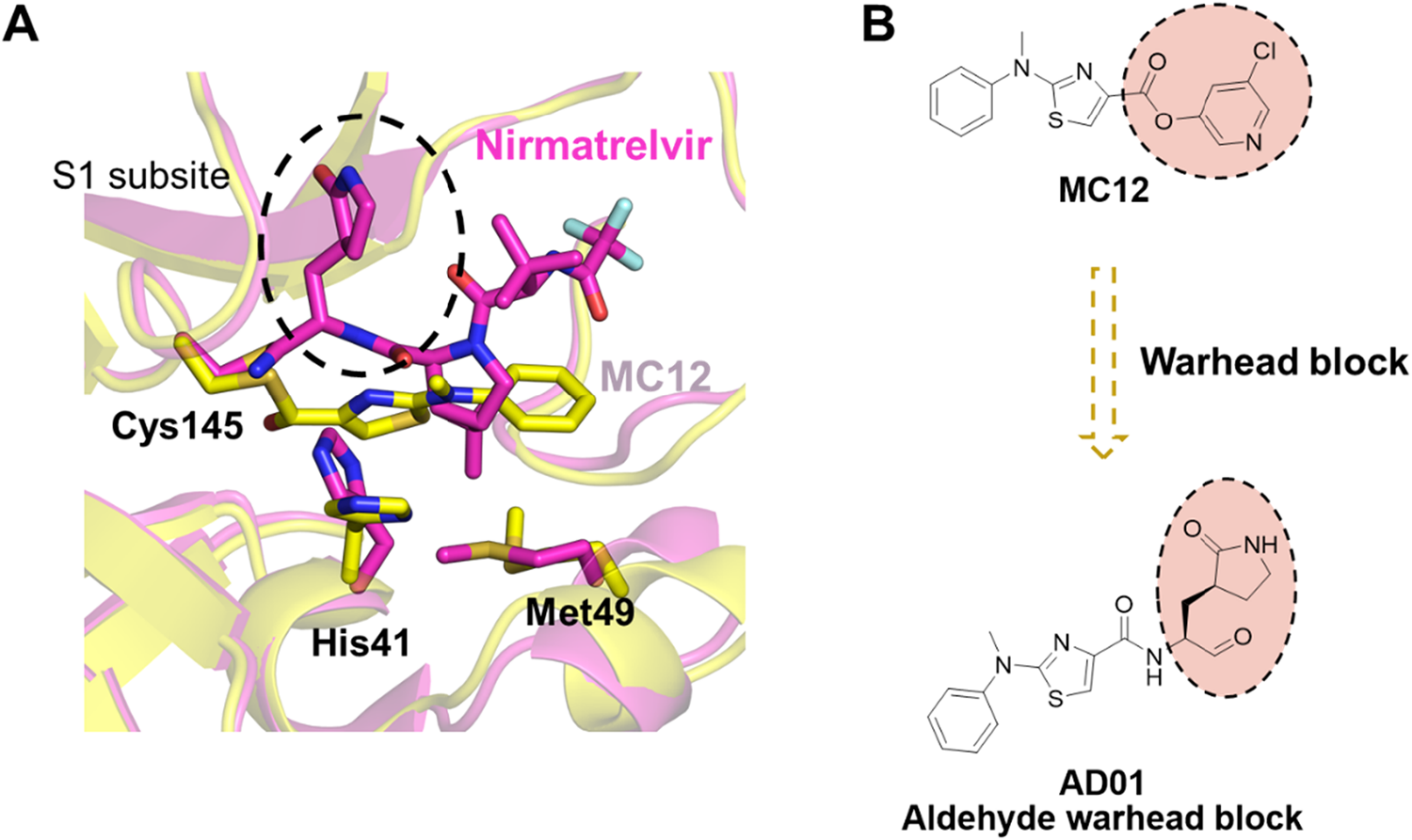
(A) Comparison of X-ray crystal structures of SARS-CoV-2 M^pro^ in complex with Nirmatrelvir (PDB ID: 8DZ2, purple) and inhibitor MC12 (PDB ID: 9M2V, yellow). The thiazole ring of MC12 stabilizes the complex via π–π stacking with His41, an interaction mode similar to that of the P2 moiety of Nirmatrelvir. Key structural differences between MC12 and Nirmatrelvir are indicated with a black dashed circle. (B) Structural modification of the warhead moiety: the pyridinyl ester warhead in MC12 was replaced with an aldehyde-based warhead to produce AD01.

The results of inhibitory evaluation indicated that compound AD01 exhibited lower inhibitory effects than MC12, but noticeable bioactivity of AD01 against SARS-CoV-2 M^pro^ was still observed (Table S1). This finding showed the critical role of the warhead group in regulating anti-SARS-CoV-2 M^pro^ efficacy. Analysis of the structural features of AD01 suggested that further structural optimization can be done by introducing groups targeting specific enzymatic subsites to improve the inhibitory effectiveness of the compounds.

Owing to their intrinsic flexibility, peptidomimetic scaffolds generally display higher aqueous solubility in comparison with nonpeptidic small molecules. Subsequent structural modifications were concentrated on merging the thiazole group with the peptidomimetic skeleton. On the basis of the substrate sequence features of the main proteases, *L*-leucine was chosen and inserted between the aldehyde segment and the thiazole moiety (Figure 4A). In addition, a bulky moiety was used to replace the thiazole group to create compound AD04 (Figure 4A). In the bioassay of inhibitory effects, compound AD04 showed diminished bioactivity compared to AD01 and AD02. This finding underscores the importance of proper fitting in the subsite of S3/4 (Figure 4B). Among this series of analogues, compound AD02 displayed the strongest inhibitory potency against SARS-CoV-2 M^pro^, implying that distinct structural characteristics at the P2 position (Figure 4A, highlighted in green) are critical for inhibitory effectiveness and binding affinity. Such findings provide important information about the ongoing structural refinement of M^pro^ inhibitors.

**Figure 4:**
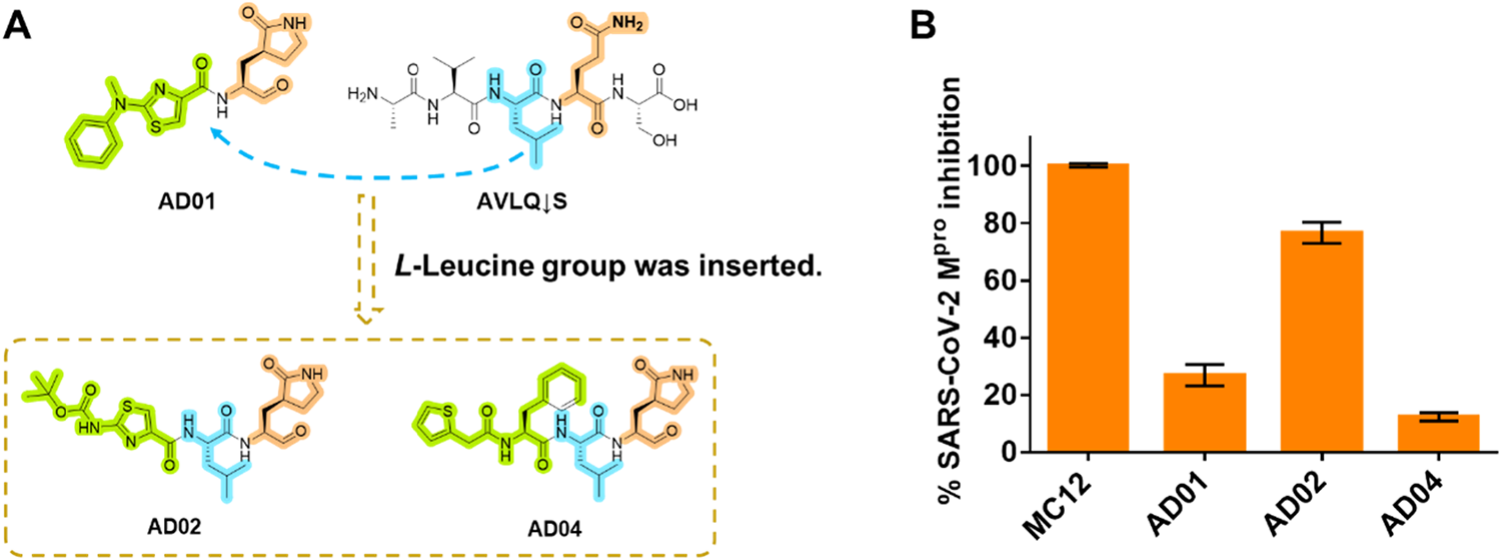
(A) Structural modification of AD01: *L*-leucine was incorporated between the aldehyde warhead and the thiazole moiety. (B) Evaluation of inhibitory activity against SARS-CoV-2 M^pro^. Compound AD02 exhibited the highest inhibitory activity against SARS-CoV-2 M^pro^, indicating that the distinct side chain of the P2 moiety contributes to enhanced binding affinity and inhibitory efficacy.

For further improvement, a 2-chlorothiazole group was used to replace the *N-tert*-BOC-2-aminothiazole in AD02 to generate the derivative AD05 (Figure 5A). Meanwhile, *L-*valine was introduced at the P3 position alongside the substitution of *N-tert*-BOC-2-aminothiazole with 2-chlorothiazole, yielding compound AD06 (Figure 5A). The result of inhibitory assays showed that the steric size of substituents at the 2-position of the thiazole ring obviously affected the overall inhibitory activity (Figure 5B). Compound AD05, which possesses a smaller P3 moiety, exhibited superior inhibitory potency when compared to AD02. This result indicates the importance of rational steric matching of the thiazole fragment. In addition, AD06 exhibited the strongest inhibition among the three compounds at 2 μM and even had a higher potency than AD02 at a lower concentration (1 μM or 500 nM). The result reveals that the incorporation of *L*-valine at the P3 position significantly enhances the inhibitory effectiveness and binding affinity (Figure 5B).

**Figure 5:**
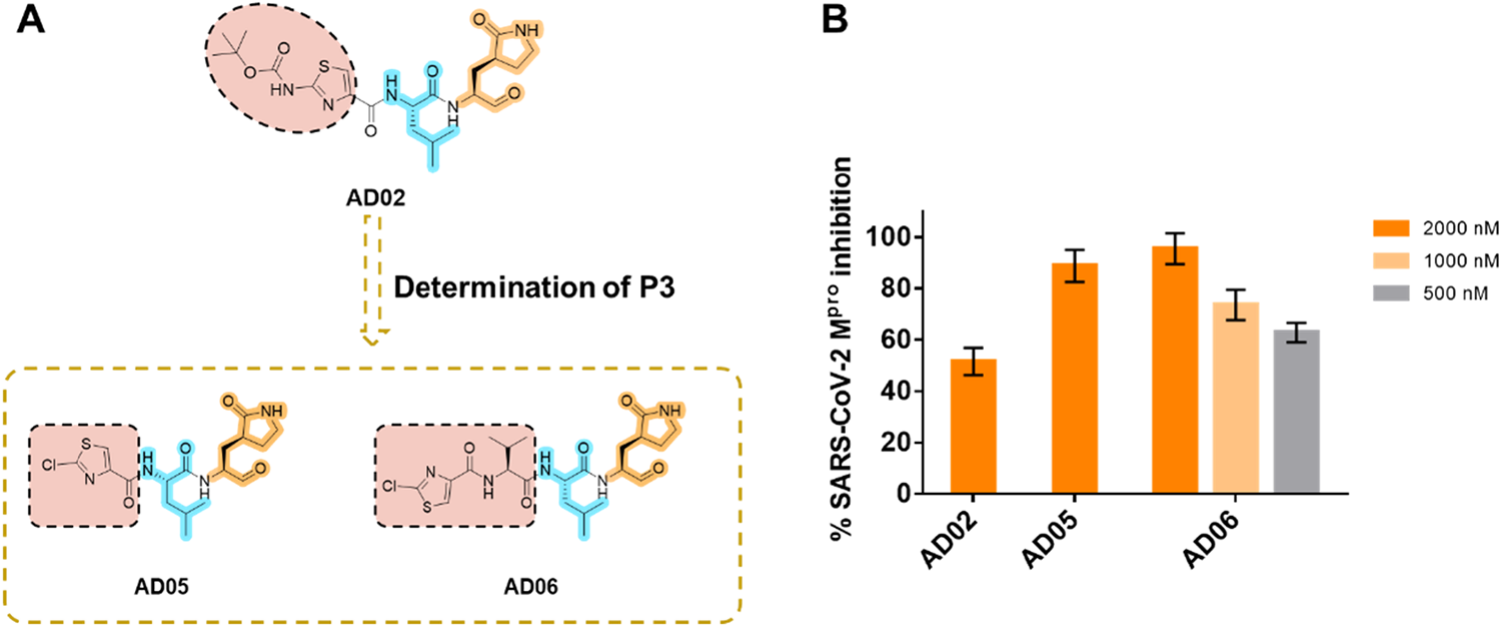
(A) Structural modification of compound AD02. (B) Evaluation of inhibitory activity against SARS-CoV-2 M^pro^. The structural size effects of the P3 and P4 moieties were investigated; *L*-valine at the P3 position enhances binding affinity and inhibitory efficacy.

To evaluate the impact of warhead types on inhibitory potency, a nitrile group was utilized to replace the aldehyde group in AD06 to generate AD06CN01 (Figure 6A). Compound CN01 is an AD02 analogue in which the P2 moiety is *L*-phenylalanine (replacing *L*-leucine in AD02), and the warhead is a nitrile (replacing the aldehyde in AD02). Additionally, the thiazole group in CN01 was replaced by an oxazole moiety to give compound CN02 while retaining the nitrile warhead (Figure 6A). Compounds CN01 and CN02 were designed to understand the contribution of the thiazole heterocycle to overall inhibitory potency. The evaluation of inhibitory activity demonstrated that the nitrile group warhead is as effective as the aldehyde. However, a reduction in the inhibitory activity of CN02 was observed after the thiazole ring was substituted with an oxazole structure (Figure 6B). These findings suggest that the chemical properties of reactive warheads and heterocyclic groups are critical determinants of the inhibitory and binding performances of AD compounds against SARS-CoV-2 M^pro^.

**Figure 6:**
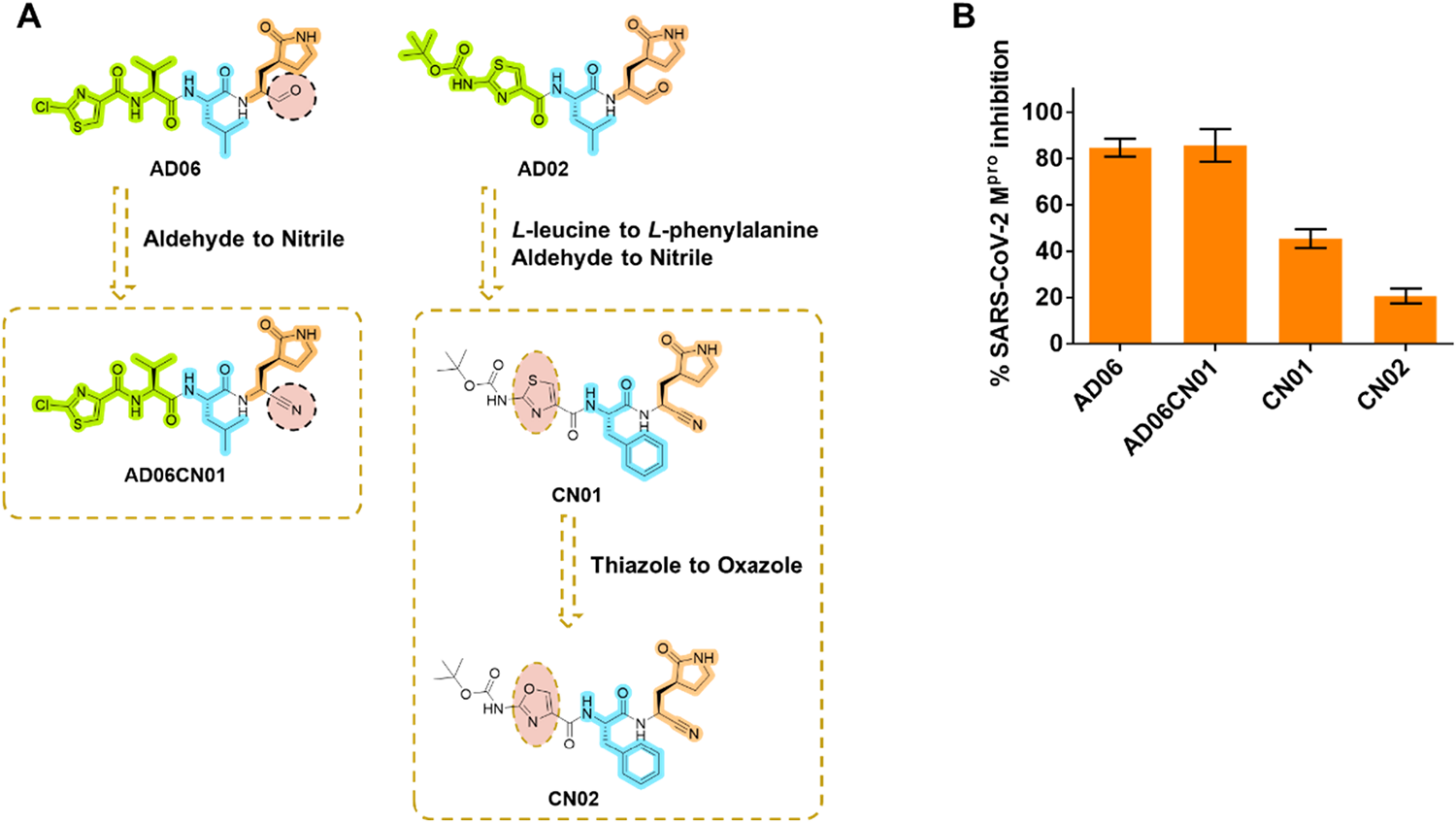
(A) Structural modification of compounds AD06, AD02, and CN01. (B) Evaluation of the inhibitory activity of the thiazole moiety and warhead on SARS-CoV-2 M^pro^. Screening results revealed that the thiazole group (CN01) as the P3 moiety displays higher inhibitory activity than the oxazole ring (CN02).

### Determination of the IC_50_ Values against SASR-CoV-2 M^pro^

Among the prepared compounds, AD05 and AD06 were chosen for the determination of the IC_50_ values against the M^pro^ of SARS-CoV-2 (Figure S1). The assessment was performed with a working enzymatic concentration of 200 nM. An IC_50_ value of approximately 306.5 ± 44.2 μM was exhibited by AD05. In contrast, a much lower IC_50_ value of 163.3 ± 43.5 nM was displayed by AD06, which is comparable to the IC_50_ value of 160.2 ± 15.1 nM exhibited by nirmatrelvir, which was used as a positive control (Table 1). Although AD06 displayed a slightly higher value of IC_50_ than the positive control, this quantitative evaluation of its inhibitory efficacy confirms that it has the potential to serve as a promising candidate for subsequent study.

**Table 1:** IC_50_ Values of the Selected Compounds against SARS-CoV-2 M^pro^

compounds	IC_50_ values (nM)
AD05	306.5 ± 44.2
AD06	163.3 ± 43.5
MC12^a^	167.4 ± 28.6
Nirmatrelvir^a^	160.2 ± 15.1

a MC12 and nirmatrelvir as positive controls.

### Crystal Structures of SARS-CoV-2 M^pro^ in Complex with AD05 and AD06

To confirm the mechanism of inhibition exerted by AD compounds against SARS-CoV-2 M^pro^, the crystal structures of the M^pro^-inhibitor complexes with AD05 (PDB ID 9M29) (Figure 7A) and AD06 (PDB ID 9M2U) (Figure 7B) were obtained. The structural data uncover the detailed interactions of AD05/AD06 within the active pocket of SARS-CoV-2 M^pro^. Both analogues are covalently anchored to the catalytic Cys145 residue and establish extensive contacts with the S1 and S2 subsites. A carbon–sulfur covalent bond is formed between the thiol group of Cys145 of SARS-CoV-2 M^pro^ and the aldehyde carbon of AD05/AD06. The oxygen atom of the aldehyde warhead can stabilize the conformation through hydrogen-bond formation with the main chain of Cys145. The (*S*)-*γ*-lactam moiety located at the P1 region of AD05/AD06 is well accommodated in the S1 subsite, stabilized by hydrogen bonding with the main chain of Phe140 and the side chain of Glu166. Moreover, the central amide group along the inhibitor backbone participates in hydrogen-bonding networks involving the main-chain residues of His164. The P2 *tert-*butyl substituent inserts deeply into the hydrophobic S2 pocket, creating π-stacking with the imidazole ring of His41 and being encapsulated by Met49 and Met165 to produce robust hydrophobic interactions. In the AD06-bound complex, the carbonyl and amino groups of Glu166 jointly fix the P3 fragment of AD06 and optimize its spatial accommodation within the binding pocket. Meanwhile, the terminal 2-chlorothiazole unit of AD06 further strengthens binding affinity via hydrophobic contacts with Pro168. Collectively, these distinct intermolecular interaction features enable AD06 to adopt a more favorable binding configuration toward SARS-CoV-2 M^pro^.

**Figure 7:**
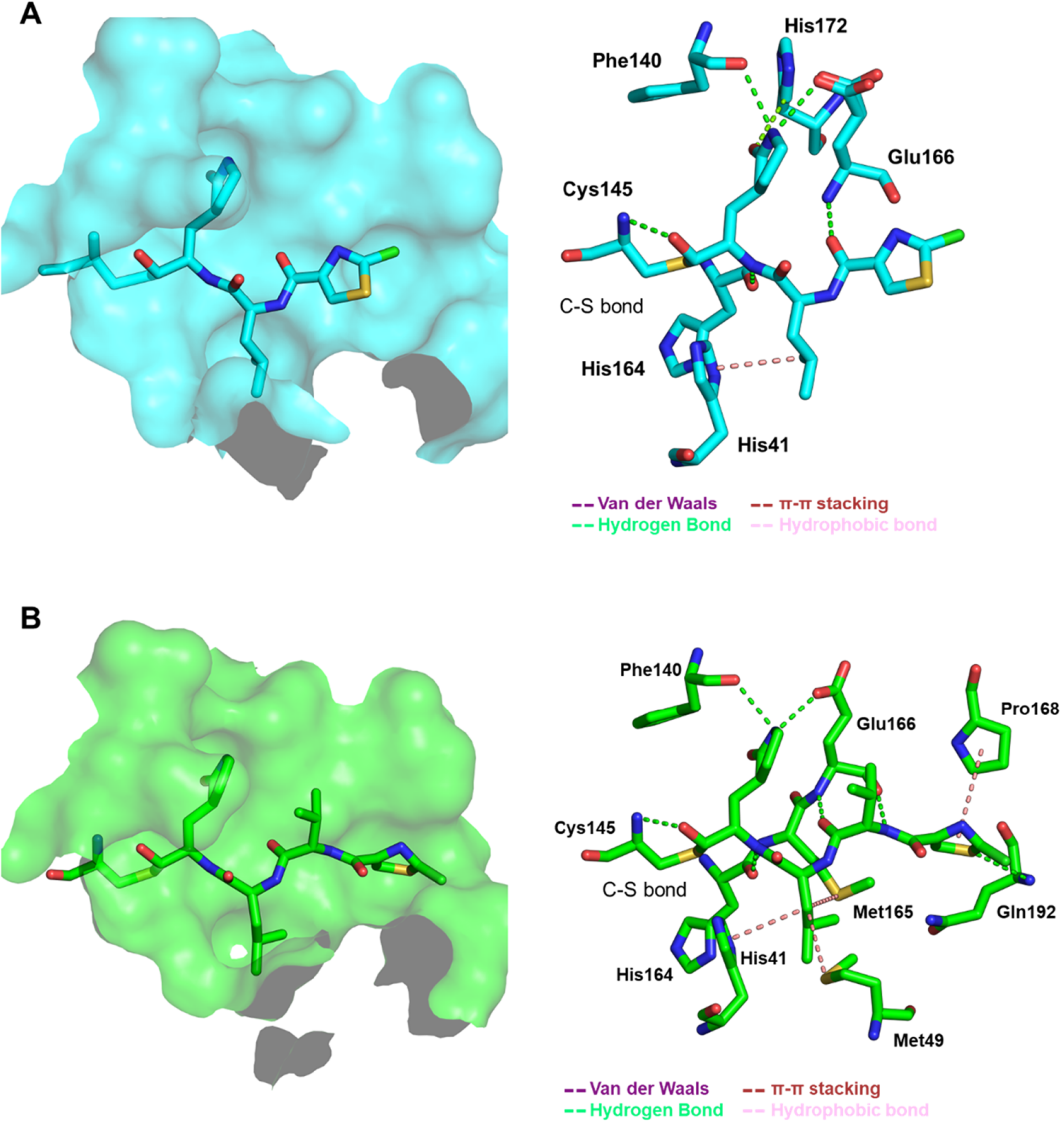
X-ray crystal structures of SARS-CoV-2 M^pro^ in complex with (A) AD05 (PDB ID: 9M29) and (B) AD06 (PDB ID: 9M2U). Both compounds fit well into the catalytic pocket of SARS-CoV-2 M^pro^.

The inhibitory mechanism mediated by AD05/AD06 is depicted in Figure S3. The thiol group of Cys145 is deprotonated by the imidazolyl of His41, followed by attack of the activated cysteine on the carbonyl carbon of AD05/AD06. This reaction results in the dysfunction of the catalytically important residue Cys145. This procedure disrupts the viral replication cycle and the entire downstream production of the viral components.

Figure 8 compares the binding modes of compound AD06 with compound AD05 (Figure 8A) and nirmatrelvir (Figure 8B). The analysis reveals that AD06 shares an identical binding mode with AD05 in the S2, S1, and S1′ subsites. Additionally, the overall binding profile of AD06 in complex with SARS-CoV-2 M^pro^ exhibited high similarity to that of nirmatrelvir observed in the crystal structure of SARS-CoV-2 M^pro^ (PDB ID: 8DZ2). However, primary differences were detected at the S1′, S2, and S3/4 pocket regions. Such distinctions are mainly attributed to divergent warhead architectures, as well as the varied steric volumes of P2 and P3 functional moieties among these inhibitors. These structural differences substantially modulate the overall binding affinity and specific binding interactions toward the target enzyme.

**Figure 8:**
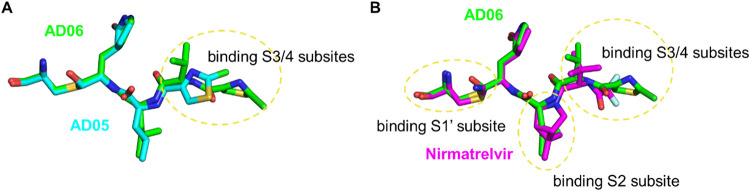
Comparison of the binding modes of (A) AD06 with AD05 and (B) AD06 with nirmatrelvir (PDB ID: 8DZ2) in SARS-CoV-2 M^pro^. The major differences between AD06 and nirmatrelvir, as well as between AD06 and AD05, are indicated with dashed circles.

### Insight into Structure-Activity Relationship

The structure of the AD compounds can be divided into five key parts: warhead, P1, P2, P3, and P4 (Figure S2). The structure-activity relationship (SAR) of the synthesized compounds was summarized to clarify the contribution of each structural fragment to their inhibitory activity against SARS-CoV-2 M^pro^. The aldehyde warhead forms a covalent bond with Cys145 residue. Substitution of this aldehyde group with a nitrile moiety maintains comparable bioactivity. At the P1 position, the S1 subsite is highly conserved and prefers a glutamine-like residue. The cocrystal structures validated that a lactam ring, as a glutamine mimetic, is essential for recognition of the S1 subsite. For the P2 position, the S2 subsite tolerates limited steric variation. The results, through comparing an aliphatic isopropyl group with an aromatic phenyl group, suggest that small, hydrophobic packing is the dominant driver at this subsite. The carbonyl group of P3 is indispensable for interaction with the main chain of Glu166, which stabilizes the bioactive conformation of the inhibitor. This hydrogen-bond interaction of the P3 carbonyl group with Glu166 is conserved across these compounds and explains why even minor modifications at this position can abolish activity. The S3/S4 subsites are more solvent-exposed and require a balance between hydrophobicity and hydrophilicity. The size, polarity, and lipophilicity of the P3/P4 fragments were systematically varied. Non-amino acid residues at P3/P4 positions are tolerated, providing flexibility for future conjugation or prodrug strategies. Incorporation of a valine moiety at the P3 position improves solubility relative to the sterically larger thiazole fragment, likely due to its smaller size and reduced hydrophobic surface area. The natural backbone of *L*-valine also offers improved compatibility with the hydrogen-bonding network around Glu166. Additionally, comparing the thiazole and oxazole rings, the thiazole exhibits higher inhibitory potency, which can be rationalized by the lower electronegativity and larger atomic radius of the sulfur atom, enhancing π-stacking polarizability with the flat, solvent-exposed surface of the S3 subsite. The reduced inhibitory activity by replacing thiazole with oxazole significantly reduces activity, indicating the importance of this heterocycle for optimal target engagement.

### Antiviral and Cytotoxic Assays of the Compounds in Vero E6

All antiviral testing procedures were carried out in the Biosafety Level 3 (BSL-3) laboratory operated by WuXi AppTec (Hong Kong) Limited. Screening assays based on cytopathic effect (CPE) serve as a fundamental experimental strategy within drug discovery research, which is widely applied to screen candidate molecules with antiviral potency against Zika virus, coronaviruses, and other pathogenic viruses. Distinct morphological alterations defined as CPE are commonly triggered in mammalian host cells after viral infection. In this CPE-based assay, the antiviral capacities of synthesized compounds against the wild-type virus of SARS-CoV-2 were tested, and their corresponding EC_50_ values were further calculated. Compound AD06, the most potent inhibitor in enzymatic screening assays, was prompted for EC_50_ evaluation. Meanwhile, compounds AD05 and MC12 were also selected for antiviral activity assays. Nirmatrelvir was applied in this study as the positive control agent. As summarized in Table S2, nirmatrelvir delivered the most potent inhibition against the virus of SARS-CoV-2. Among the designed compounds, AD05 (EC_50_ = 3.22 ± 0.61 μM) demonstrated nearly eight times stronger antiviral activity than AD06 (EC_50_ = 25.58 ± 0.71 μM), despite showing lower enzymatic inhibitory activity (Figure S4). Compound AD06 showed moderate antiviral activity, while the EC_50_ value of MC12 against SARS-CoV-2 was more than 100 μM. These results suggest that thiazole-based peptidomimetic compounds are potential SARS-CoV-2 antiviral agents.

The apparent disconnect between the enzymatic and cellular activities of AD05/AD06 can be rationalized by their distinct physicochemical properties. To elucidate the factors behind their distinct antiviral activities, the key parameters log *P*, topological polar surface area (TPSA), and number of hydrogen-bond donors were calculated using the free online tool SwissADME (http://www.swissadme.ch/). Log *P* is the logarithm (base 10) of the organic-to-aqueous phase concentration ratio. The value of log *P* indicates the amphiphilic balance of a drug. An optimal log *P* between 0 and 5 ensures sufficient hydrophilicity for dissolution in blood and sufficient lipophilicity to cross lipid membranes, with lower values favoring aqueous solubility. The calculated log *P* values for AD05 and AD06 were 1.78 and 2.14, respectively. The lower log *P* of AD05 suggests better solubility in the culture medium and a higher effective extracellular concentration, which might lead to a higher intracellular concentration and consequently stronger anti-SARS-CoV-2 activity. In comparison, AD06 exhibits properties that impede cellular efficacy according to Veber’s rules. The higher TPSA (174.6 *vs* 145.5 Å^2^) and more hydrogen-bond donors (4 *vs* 3) of AD06 predict poor passive membrane permeability. Additionally, the higher log *P* of AD06 predicts greater binding to serum albumin, reducing the free concentration in the medium. These mechanisms collectively provide a convergent explanation for the weaker antiviral activity of AD06 despite its superior enzymatic inhibition.

The cytotoxicity of the target compounds was evaluated under identical culture conditions without virus infection (Table S2). Neither AD06 nor AD05 showed obvious cytotoxicity toward Vero E6 cells within the range of the tested concentration (CC_50_ > 100 μM). The results of strong antiviral efficacy and negligible cytotoxicity suggest that AD05 is a promising candidate for the further development of safe and effective anti-SARS-CoV-2 agents.

## Conclusions

In this study, we have systematically explored the structure–activity relationship (SAR) of a series of AD compounds targeting the main protease (M^pro^) of SARS-CoV-2. Our findings provide valuable insights into the molecular interactions that drive the inhibitory activity of these compounds and highlight the key structural features that contribute to their efficacy. Through detailed crystallographic analysis and inhibitory activity assays, we have demonstrated that the aldehyde and nitrile groups of thiazole-based peptidomimetics are both effective as warheads, forming covalent bonds with the catalytic Cys145 residue. The lactam ring at the P1 position is important for identification by the S1 subsite. The S2 subsite prefers hydrophobic groups at the P2 position. The carbonyl group at the P3 position plays a key role in stabilizing the active conformation of the inhibitor through interactions with Glu166. In addition, the thiazole group at the P3 position displays greater inhibitory activity than the oxazole group, highlighting the critical role of the heterocyclic fragment in binding to the SARS-CoV-2 M^pro^.

Our results highlight the potential of both AD05 and AD06 as valuable lead compounds for further structural optimization. AD06 exhibited enzymatic inhibitory potency against SARS-CoV-2 M^pro^ (IC_50_ = 163.3 ± 43.5 nM) as strongly as nirmatrelvir. Detailed binding mode analysis revealed that AD06 accommodates the active site in a favorable conformation in the cocrystal structure of SARS-CoV-2 M^pro^. In contrast, AD05 exhibited superior antiviral activity in the CPE assays against wild-type SARS-CoV-2 (EC_50_ = 3.22 ± 0.61 μM) compared to AD06 (EC_50_ = 25.58 ± 0.71 μM), despite showing lower enzymatic inhibition (IC_50_ = 306.5 ± 44.2 nM). Both compounds exhibited low cytotoxicity in Vero E6 cells. The discrepancy between the enzymatic and antiviral activity of AD06 may be attributed to suboptimal cellular permeability, a common challenge in drug discovery that can be addressed through further medicinal chemistry efforts. These compounds represent two complementary starting points for optimization. Future research could concentrate on improving the cellular permeability of AD06-based compounds while maintaining their enzymatic potency, and on enhancing the enzymatic affinity of AD05-based compounds to potentially yield even more potent antiviral agents with proper pharmacokinetic profiles. In conclusion, the results obtained in this work establish a solid foundation for the continued optimization of AD compounds as potent inhibitors against SARS-CoV-2 M^pro^. The insights gained from this study will be instrumental in guiding the research of novel antiviral agents to combat SARS-CoV-2 and related coronaviruses.

## Methods

### Materials and Equipment

All commercial reagents and solvents were used directly as received with no additional purification. Column chromatography was carried out on 300 mesh silica gel. Reactions were monitored via thin-layer chromatography (TLC) on F254 fluorescent silica gel plates, with spot visualization achieved under ultraviolet (UV) light. All candidate compounds in this study were chemically synthesized in-house. Enzymatic kinetic parameters were determined with a TECAN Freedom EVO 100 platform. The fluorogenic peptide substrate MCA-AVLQ↓SGFR-Lys­(DNP)-Lys-NH_2_ was acquired from GL Biochem (Shanghai, China). ^1^H NMR spectra were recorded on either a JEOL ECZ500R 500 MHz NMR or a Bruker Advance-III 400 MHz FT-NMR spectrometer. Purified fractions were vacuum-dried and subsequently redissolved in 600 μL of DMSO-*d*_6_ within NMR tubes for spectral analysis. High-resolution mass spectrometry (HRMS) was applied to confirm the structural identity of all final products. The purified end products were dissolved in HPLC-grade methanol, and the resulting solution (approximately 1 μM) was infused into an Agilent 6540 LC-ESI-Q-TOF HRMS system at a constant flow rate of 200 μL/min. Detailed experimental procedures are provided in the Supporting Information.

## Supplementary Material



## Notes

NMR spectra and HRMS data for the designed compounds, together with the determination of EC_50_ values of compounds AD05 and AD06 against SARS-CoV-2 βCoV/Wuhan/WIV04/2019 virus strain in Vero E6 cell line and cytotoxic assay of compounds AD05 and AD06 in Vero E6 cell line (Figure S1); the factors and coordinates for the SARS-CoV-2 M^pro^ with AD05 and AD06 with a covalent linkage have been deposited in the PDB with the ID: 9M29 and 9M2U, respectively (PDF)
