# Supplementary material for: Design, Synthesis, and Evaluation of Novel Thiazole-Based Peptidomimetic Compounds as Potent SARS-CoV‑2 Main Protease Covalent Inhibitors
Source: ACS Infect Dis. 2026 May 20;12(6):1915–23. doi: 10.1021/acsinfecdis.6c00019 (PMC13270522; doi:10.1021/acsinfecdis.6c00019)
Supplement: Supplementary file 1 [file id6c00019_si_001.pdf]

Supporting Information

Design, Synthesis, and Evaluation of Novel Thiazole-Based Peptidomimetic Compounds as Potent SARS-CoV-2 Main Protease Covalent Inhibitors

Weile YIN, Wai-Po KONG, Siu-Lun LEUNG, Zhiguang LIANG, Yu Wai CHEN, Kwok-Yin WONG[1]

Department of Applied Biology and Chemical Technology, The Hong Kong Polytechnic University, Hung Hom, Hong Kong, China

Contents

Inhibitory results of MC12 and AD01 against SARS-CoV-2 M^{pro} 3

Results of CPE assay 4

IC_{50} evaluation of selected compounds 5

Structural components of AD compounds 6

Mechanism of inhibition 7

Antiviral assay 8

Cocrystal structures 9

METHODS 12

Materials and equipment. 12

[1] Corresponding author.

Cloning, expression, and purification of the recombinant SARS-CoV-2 M^{pro}. 12
Measurement of SARS-CoV-2 M^{pro} biochemical activity. 14
Evaluation of IC_{50} of compounds against SARS-CoV-2 M^{pro}. 15
Crystallization, data collection and structure determination. 16
Chemistry 18
^{1}H NMR and high-resolution mass spectra 38

Inhibitory results of MC12 and AD01 against SARS-CoV-2 M^{pro}

Table S1. Inhibitory activities of MC12 and AD01 against SARS-CoV-2 M^{pro}.

Compounds	% inhibition at 2 μM
MC12 [a]	99.8 ± 0.7
AD01	30.9 ± 2.7

[a] Compound MC12 served as the positive control. The designed compound AD01 exhibited an inhibitory rate of approximately 30.9% against SARS-CoV-2 M^{pro}, demonstrating weaker inhibitory activity than that of MC12.

Results of CPE assay

Table S2. Results of CPE assay of AD05 and AD06 against SARS-CoV-2 βCoV/Wuhan/WIV04/2019 virus strain in Vero E6 cell line (conducted by WuXi AppTech).

**Compounds**	**EC_{50} (μM)**	**CC_{50} (μM)**	**SI (CC_{50}/EC_{50})**
AD05	3.22	> 100	>30.64
AD06	25.58	> 100	>3.87
MC12	>100	N/D[bb]	N/A[bbb]
Nirmatrelvir[b]	0.077	> 5	>1393.34

[b] Nirmatrelvir as positive control. [bb] N/D: not detected. [bbb] N/A: not applicable. Although Although AD06 exhibited stronger enzymatic inhibition against SARS-CoV-2 M^{pro} than AD05, AD05 displayed approximately 8-fold higher antiviral activity than AD06.

IC$_{50}$ evaluation of selected compounds

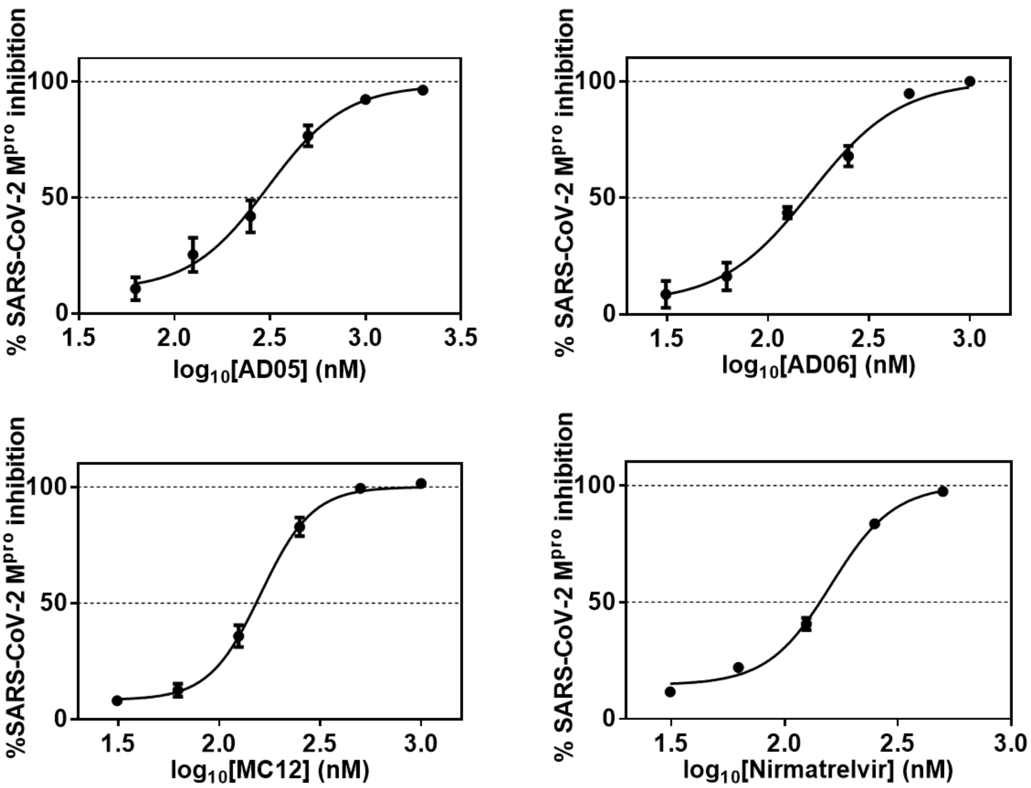

Figure S1. IC$_{50}$ evaluation of selected compounds against SARS-CoV-2 M^{pro}. Compounds MC12 and nirmatrelvir served as positive controls.

Structural components of AD compounds

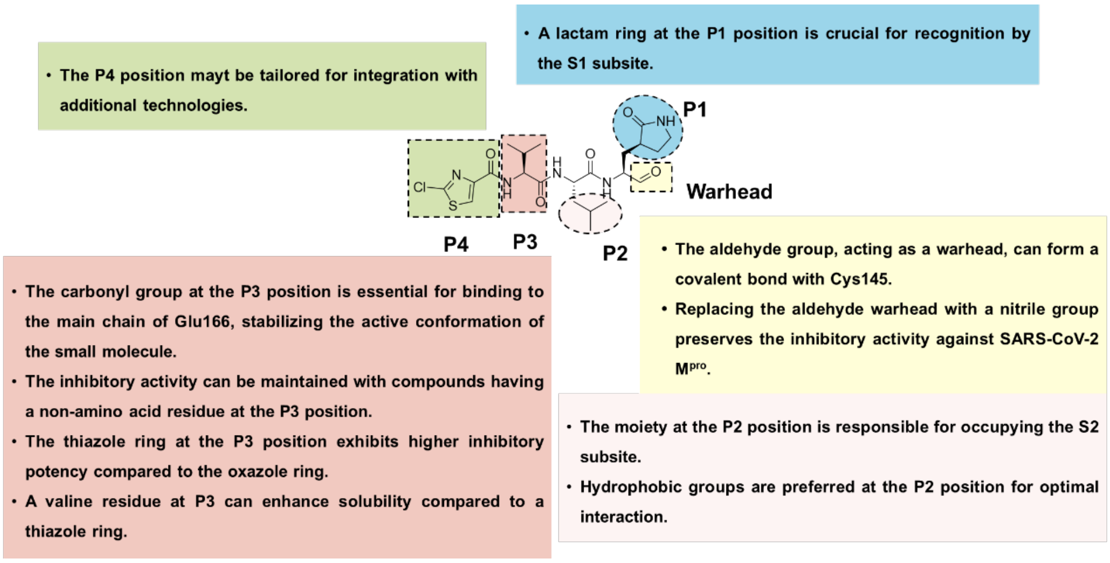

Figure S2. Five Components of AD Compounds.

Mechanism of inhibition

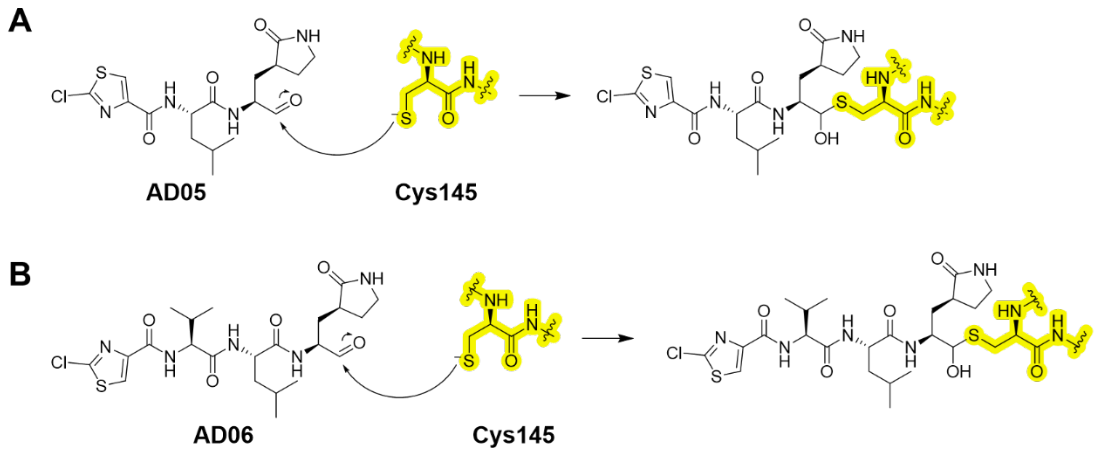

Figure S3. Mechanism of inhibition of SARS-CoV-2 M^{pro} by (A) AD05 and (B) AD06.

Both AD05 and AD06 covalently bind to Cys145 of SARS-CoV-2 M^{pro}.

Antiviral assay

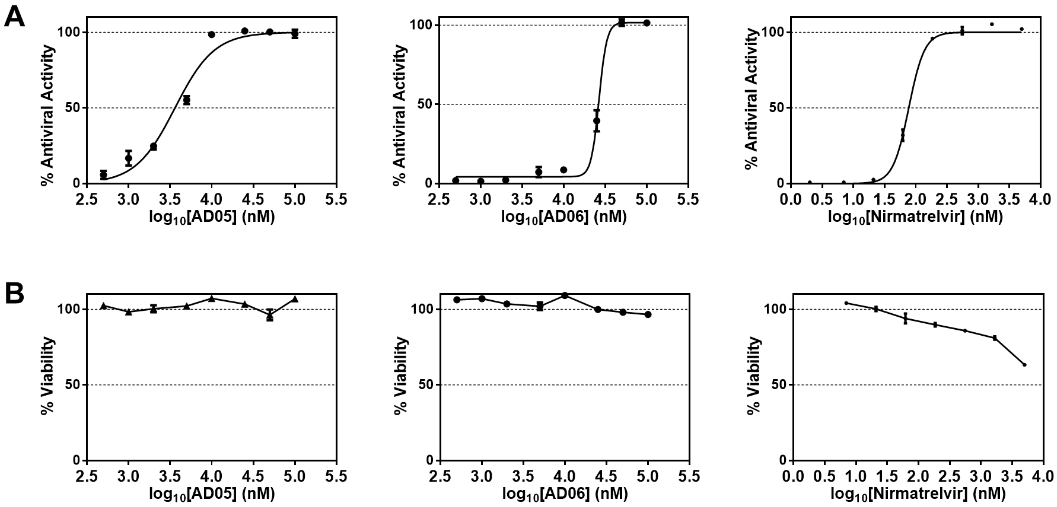

Figure S4 A) The determination of EC50 values of compounds AD05 and AD06 against SARS-CoV-2 βCoV/Wuhan/WIV04/2019 virus strain in Vero E6 cell line; B) Cytotoxic assay of compounds AD05 and AD06 in Vero E6 cell line. (Nirmatrelvir as a positive control) (conducted by the Biosafety Level 3 (BSL-3) laboratory of WuXi AppTec (Hong Kong) Limited).

Cocrystal structures

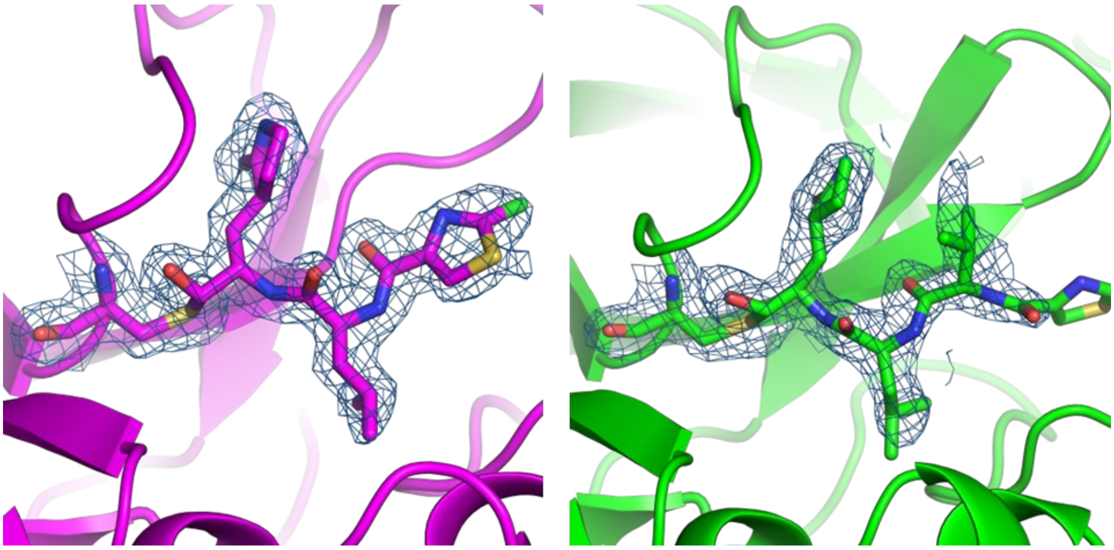

Figure S5. Electron density map of the protein-substrate complexes. The AD05 (left hand side, purple, PDB ID: 9M29) and AD06 (right hand side, green, PDB ID: 9M2U) compounds covalently bound to Cys145 in M^{pro} are shown with a $2mF_o - DF_c$ electron density map contoured at 1 σ (blue mesh). The main-chain and side-chain of Cys145 are illustrated in ball and stick style with the corresponding inhibitor colour.

Table S3. Data collection and refinement statistics. The values in parentheses are for the highest resolution shell (2.02 - 1.97 Å).

	Mpro-AD05	Mpro-AD06
**PDB code**	9M29	9M2U
**Data Collection**		
Space group	I121	I121
Cell dimensions		
a, b, c (Å)	51.52, 80.14, 88.97	46.15, 52.89, 110.62
α, β, γ (°)	90.00, 97.47, 90.00	90.00, 101.80, 90.00
Wavelength (Å)	1.54184	1.54184
Resolution	29.26-9.01 (2.02-1.97)	27.16-9.01 (2.02-1.97)
Completeness	94.1% (97.9%)	95.3% (94.2%)
R_{merge} (%)	3.1% (14.4%)	6.8% (82.2%)
I/σ[a]	4.06(1.96)	1.12(1.96)
**Refinement**		
Resolution (Å)	23.68-1.97	23.77-1.97
No. of reflections	25783	18445
R_{work}/R_{free}	0.176/0.216	0.228/0.316
**B factor (Å2)**		
Protein	23.74	34.08
Ligand	25.86	25.68
Water	26.66	24.88

**RMSD**		
Bond length (Å)	0.96	1.04
Bond angles (°)	1.53	1.75
**Ramachandran plot (%)**		
Favoured	96	93
Allowed	4	6

METHODS

Materials and equipment.

All commercial reagents and solvents were used directly as received with no additional purification. Column chromatography was carried out on 300-mesh silica gel. Reaction progress was tracked via thin-layer chromatography (TLC) on F254 fluorescent silica gel plates, with spot visualization achieved under ultraviolet (UV) light. All candidate compounds in this study were chemically synthesized in-house. Enzymatic kinetic parameters were determined with a TECAN Freedom EVO 100 platform. The fluorogenic peptide substrate MCA-AVLQ↓SGFR-Lys(DNP)-Lys-NH2 was acquired from GL Biochem (Shanghai, China). The ^{1}H and ^{13}C NMR spectra were recorded on either a Bruker Advance-III 400 MHz FTNMR spectrometer or a Jeol ECZ500R 500 MHz NMR instrument. Purified fractions were vacuum-dried and subsequently redissolved in 600 μL of DMSO-d_6 within NMR tubes for spectral analysis. High-resolution mass spectrometry (HRMS) was applied to confirm the structural identity of all final products. The purified end products were dissolved in HPLC-grade methanol, and the resulting solution (approximately 1 μM) was infused into an Agilent 6540 LC-ESI-Q-TOF HRMS system at a constant flow rate of 200 μL/min.

Cloning, expression, and purification of the recombinant SARS-CoV-2 M^{pro}.

The method of cloning and producing recombinant SARS-CoV-2 M^{pro} followed the protocol published previously [1]. The full-length gene that encodes SARS-CoV-2 M^{pro}

(NC_045512) was cloned into pGEX-6P-1 (Bio-Gene). At the *N*-terminus, the SARS-CoV-2 M^{pro} construct contains a GST-tag followed by the M^{pro} cleavage site (SAVLQ↓SGFRK; the arrow indicates the cleavage site). At the C-terminus, the construct codes for a modified PreScission cleavage site connected to a His6-tag. The verified SARS-CoV-2 M^{pro} construct was transformed into the *Escherichia coli* (*E. coli*) strain BL21-Gold (DE3) (Novagen). The transformed cells were incubated at 37°C in 2x Yeast Extract Tryptone (2xYT) medium with ampicillin (100 μg/mL) until the OD_{600} reach 0.8. Protein induction was then induced by 1 mM isopropyl-D-thiogalactoside (IPTG) at 16°C for 16 hours before harvested by centrifugation (10,000 x g, 4°C). The pellets were resuspended in 30 mL of buffer A (20 mM Tris, 150 mM NaCl, pH 7.8) and then lysed by sonication on ice. The lysate was clarified by centrifugation at 39,200 x g at 4°C for 30 min. The supernatant was filtered by 0.2 μm sterile filter (Millipore) and applied to a 5 mL HisTrap column (GE Healthcare). The HisTrap column was washed with 10 column volumes (CVs) of buffer A followed by elution with gradient increasing of buffer B (20 mM Tris, 150 mM NaCl, 500 mM imidazole, pH 7.8) over 20 CVs. The fractions containing the target protein were pooled and mixed with PreScission protease (GenScript) at a molar ratio of 5:1 and dialyzed into buffer C (20 mM Tris, 150 mM NaCl, 1 mM DTT, pH 7.8) at 4°C overnight. The PreScission-treated M^{pro} was applied to GSTtrap FF (GE Healthcare) and HisTrap column (GE Healthcare)to remove the GST-tagged PreScission protease and any uncleaved protein. The tag-free M^{pro} protein was further purified by HiTrap Q FF column (GE Healthcare) equilibrated with buffer D (20 mM Tris, 1 mM DTT, pH 8.0) and eluted with linear

gradient of buffer E (20 mM Tris, 1 M NaCl, 1 mM DTT, pH 7.8) with over 20CVs. Fractions containing the target protein were then buffer exchanged into buffer F(20 mM Tris, 150 mM NaCl, 1 mM EDTA, 1 mM DTT, pH 7.8). The quality of the purified M^{pro} was checked by sodium dodecyl-sulfate polyacrylamide gel electrophoresis (SDS-PAGE).

Measurement of SARS-CoV-2 M^{pro} biochemical activity.

The *in vitro* biochemical activity of the recombinant SARS-CoV-2 M^{pro} was measured as previously described [2] with modifications. The fluorogenic peptide substrate was purchased from GL Biochem in Shanghai. The purified SARS-CoV-2 M^{pro} in the buffer (20 mM Tris, 150 mM NaCl, 1 mM EDTA, 1 mM DTT, pH 7.8) was preincubated with compounds in 100% DMSO for 10 minutes at 37°C. After the fluorogenic peptide substrate in Dimethyl sulfoxide (DMSO)/buffer (20 mM Tris, 150 mM NaCl, 1 mM EDTA, 1 mM DTT, pH 7.8) (v/v = 1/1) was added to each well to achieve final concentrations of SARS-CoV-2 M^{pro}, compounds, and the fluorogenic peptide substrate at 200 nM, 150 nM, and 20 μM, respectively, fluorescence signal (excitation wavelength: 320 nm; emission wavelength: 405 nm) was continuously measured on a plate reader for 5 minutes to analyze the inhibitory activity of the compounds. For calculations, a 100% active enzyme was assumed. The experimental errors were estimated from triplicate measurements.

The inhibitory activity of the compounds was expressed as **% Inhibition** and was calculated using the following formula:

Inhibition (%) = (1 - Raw data $_{compound}$ */ Average* $_{DMSO}$*)*100*

Where:

Raw data $_{compound}$ *is the value of fluorescence intensity measured in the wells treated with the compound.*

Average $_{DMSO}$ *is the average value of fluorescence intensity measured in the wells treated with DMSO.*

Evaluation of IC_{50} of compounds against SARS-CoV-2 M^{pro}.

Inhibition of the biochemical activity of the recombinant wild-type SARS-CoV-2 M^{pro} was quantified as previously described [2], with modifications. A serially diluted solution of the tested compounds was prepared in 100% DMSO. This serially diluted solution of compounds was then added to a 96-well plate, followed by the addition of the protease to achieve a final concentration of SARS-CoV-2 M^{pro} at 100 nM. After incubating SARS-CoV-2 M^{pro} with the compounds for 10 minutes at 37°C, the fluorogenic peptide substrate was added to each well to a final concentration of 20 µM. Fluorescence signal (excitation wavelength: 320 nm; emission wavelength: 405 nm) was continuously measured on a plate reader for 5 minutes. For calculations, a 100% active enzyme was assumed [2]. The experimental errors were estimated from triplicate measurements. The inhibitory activity of the compounds was expressed as % Inhibition and calculated using the formula provided. IC_{50} values were calculated using GraphPad

Prism software (Version 8.0) with the equation log(inhibitor) vs. response with variable slope.

Crystallization, data collection and structure determination.

The protein-substrate complexes were generated by mixing SARS-CoV-2 M^{pro} with the AD05 and AD06, respectively, in a 1:10 molar ratio overnight at 4 °C. The complexes were then concentrated to 10 mg/mL and crystallized by hanging drop vapor diffusion method at 20 °C. Plate-like crystal of SARS-CoV-2 M^{pro} in complex with AD05 was grown with well buffer containing 0.1 M Bis-Tris pH 5.8, 30% (v/v) polyethylene glycol (PEG) 3350 at a 1:2 ratio. For the complex with AD06, plate-like crystal appeared in the crystallization condition comprised of 0.1 M Bis-Tris pH 6.5, 18% (v/v) PEG 3350 at a 1:2 ratio. The crystals were cryoprotected in the same crystallization buffer with an additional 10% DMSO.

The diffraction data were collected inhouse on a Rigaku MicroMax-007 HK generator with an Dectris PILATUS3 R 200K detector at 100 K. The diffraction images were processed with IMOSFLM [3],and data were reduced by AIMLESS [4] from the CCP4 suite [5]. The structure was determined by molecular replacement with Phaser [6] in CCP4 using the native SARS-CoV-2 M^{pro} structure (PDB ID: 7K3T)[7] as a search model. The output model was subsequently subjected to iterative cycles of manual adjustment with Coot [8] and refinement with REFMAC [9]. The inhibitors were built according to the omit map along with the refinement cycle.

Antiviral assay. Assays of antivirus and cytotoxicity in Vero E6 were conducted by the collaborator biosafety level 3 (BSL-3) lab of WuXi AppTec (HongKong) Limited. Vero E6 cells were provided by the Wuhan Institute of Virology and maintained in Dulbecco′s Modified Eagle Medium (DMEM, Gibco#C11995500BT) supplemented with 10% fetal bovine serum (FBS) and 1% penicillin-streptomycin (PS). DMEM supplemented with 2% FBS and 1% PS was used as the assay medium. The SARS-CoV-2 βCoV/Wuhan/WIV04/2019 strain was provided by the Wuhan Institute of Virology, Chinese Academy of Sciences. The preservation number is CSTR: 16533.06. IVCAS 6.7512.

Vero E6 cells were seeded at a density of 1×10^4 cells per well in a black 96-well plate and incubated at 37°C and 5% CO_2 overnight. The next day, the supernatant was replaced with medium containing serially diluted compounds (6 points, 3-fold dilutions, in duplicate wells) and 2 μM CP-100356. Cells were then infected with the SARS-CoV-2 βCoV/Wuhan/WIV04/2019 strain at a multiplicity of infection (MOI) of 0.005. The final concentration of DMSO in the cell culture was 0.5%. The resulting cultures were incubated at 37°C and 5% CO_2 for an additional 4 days until virus infection in the virus control (cells infected with the virus, without compound treatment) displays significant CPE. The cell control (without virus infection or compound treatment) and virus control (with virus infection, without compound treatment) were tested in parallel. The CPE was measured using CellTiter Glo, following the manual of the manufacturer. The

antiviral activity of compounds was calculated based on the protection of the virus-induced CPE at each concentration, normalized by the virus control.

The cytotoxicity of compounds was assessed under the same conditions but without virus infection, in parallel. Cell viability was measured using CellTiter Glo, following the manual of the manufacturer.

The antiviral activity and cytotoxicity of compounds were expressed as % Inhibition and % Viability, respectively, and calculated using the formulas below:

*Inhibition (%) = (Raw data $_{CPD}$ - Average $_{VC}$) / (Average $_{CC}$ - Average $_{VC}$)*100*

*Viability (%) = (Raw data $_{CPD}$ - Average $_{MC}$) / (Average $_{CC}$ - Average $_{MC}$)*100*

Where:

***Raw data** $_{CPD}$ is the values of the sample-treated wells.*

***Average** $_{VC}$ is the average value of the virus control.*

- ***Average** $_{CC}$**:** average value of the cell control*

EC_{50} and CC_{50} values were calculated using GraphPad Prism software (Version 8.0) with the equation log(inhibitor) vs. response with variable slope.

Chemistry

The synthesis of AD01 leveraged an intermediate from the MC12 preparation process, specifically 2-(methyl(phenyl)amino)thiazole-4-carboxylic acid, as the starting material. For the amide synthesis with methyl (*S*)-2-amino-3-((*S*)-2-oxopyrrolidin-3-

yl)propanoate, *N,N,N′,N′*-tetramethylchloroformamidinium hexafluorophosphate (TCFH) was used with *N*-methylimidazole (NMI) as the catalyst [10]. The intermediates were reduced to alcohols and subsequently oxidized using Dess-Martin Periodinane [11] to obtain the final aldehydes (Scheme S1).

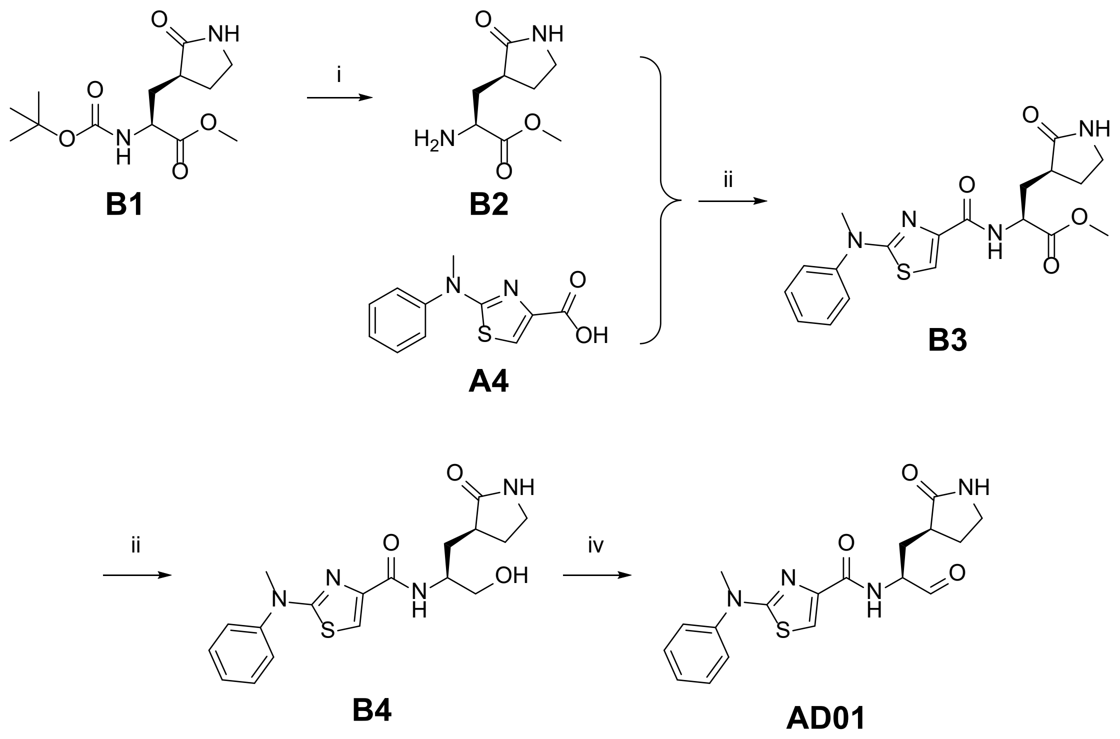

Scheme S1. Synthetic route to AD01: (i) 6 M HCl in 1,4-dioxane, room temperature, overnight; (ii) *N,N,N′,N′*-tetramethylchloroformamidinium hexafluorophosphate (TCFH), *N*-methylimidazole (NMI), acetonitrile, room temperature, overnight, 70%; (iii) $NaBH_4$, THF, room temperature, overnight, 90%; (iv) Dess-Martin Periodinane, room temperature, overnight, 76%.

The synthetic routes for AD02, AD04, AD05 and AD06 share initial steps, particularly in the synthesis of certain intermediates. The deprotection of (*S*)-2-((*tert*-

butoxycarbonyl)amino)-3-((*S*)-2-oxopyrrolidin-3-yl)propanoate was facilitated by 6 M HCl in 1,4-dioxane. Except preparation of the amide with methyl (*S*)-2-amino-3-((*S*)-2-oxopyrrolidin-3-yl)propanoate, other amides were synthesized under the catalysis of *N,N*-Diisopropylethylamine (DIPEA) and 2-(7-Azabenzotriazol-1-yl)-*N,N,N′,N′*-tetramethyluronium hexafluorophosphate (HATU). The products were then hydrolyzed to yield the corresponding carboxylic acids. For the amide synthesis with methyl (*S*)-2-amino-3-((*S*)-2-oxopyrrolidin-3-yl)propanoate, *N,N,N′,N′*-tetramethylchloroform-amidinium hexafluorophosphate (TCFH) was used with *N*-methylimidazole (NMI) as the catalyst [10]. The intermediates were reduced to alcohols and subsequently oxidized using Dess-Martin Periodinane [11] to obtain the final aldehydes (Scheme S2).

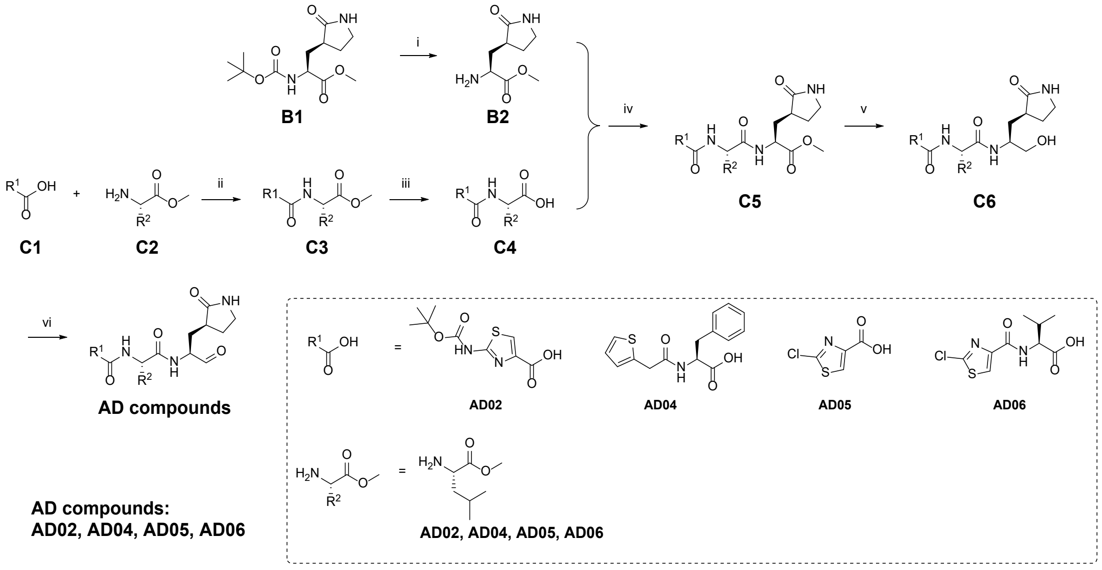

Scheme S2. Synthetic routes to AD02, AD04, AD05 and AD06: (i) 6 M HCl in 1,4-dioxane, room temperature, overnight; (ii) 2-(7-Azabenzotriazol-1-yl)-*N,N,N′,N′*-tetramethyluronium hexafluorophosphate (HATU), Triethylamine (TEA), room temperature, overnight, 70%; (iii) 0.8 M aqueous LiOH, THF, room temperature, 2 h,

80%; (iv) *N*,*N*,*N'*,*N'*-tetramethylchloroformamidinium hexafluorophosphate (TCFH), *N*-methylimidazole (NMI), acetonitrile, room temperature, overnight, 70%; (v) $NaBH_4$, THF, room temperature, overnight, 90%; (vi) Dess-Martin Periodinane, room temperature, overnight, 70%.

Procedures for the chemical synthesis of AD01. *2-(Methyl(phenyl)amino)-N-((S)-1-oxo-3-((S)-2-oxopyrrolidin-3-yl)propan-2-yl)thia zole-4-carboxamide (AD01).* Methyl (*S*)-2-((*tert*-butoxycarbonyl)amino)-3-((*S*)-2-oxopyrrolidin-3-yl)propanoate (1.0 g. 3.5 mmol) was dissolved in 10 mL of Dichloromethane, and then HCl (9 mL, 4 M in dioxane) was added. The reaction mixture was stirred at ambient temperature for 12 hours, and the mixture was concentrated under reduced pressure to obtain a white solid, methyl (*S*)-2-amino-3-((*S*)-2-oxopyrrolidin-3-yl)propanoate, which could be used for the following step without further purification.

To a solution of 2-(methyl(phenyl)amino)thiazole-4-carboxylic acid (0.23 g, 1 mmol) in acetonitrile, *N,N,N',N'*-tetramethylchloroformamidinium hexafluorophosphate (TCFH, 0.27 g, 1 mmol), *N*-methylimidazole (NMI, 0.32 g, 4 mmol), and methyl (*S*)-2-amino-3-((*S*)-2-oxopyrrolidin-3-yl)propanoate (0.18 g, 1 mmol) were added, followed by stirring at room temperature. After stirring overnight, the reaction solution was cooled to room temperature, and a solid formed. The solution was washed with a saturated aqueous NaCl solution and extracted with Ethyl acetate (10 mL × 3). The organic phase was dried with sodium sulfate and concentrated under reduced pressure

to afford the pale-yellow solid, methyl (*S*)-2-(2-(methyl(phenyl)amino)thiazole-4-carboxamido)-3-((*S*)-2-oxopyrrolidin-3-yl)propanoate, with a yield of 0.27 g (70%).

To a solution of Methyl (*S*)-2-(2-(methyl(phenyl)amino)thiazole-4-carboxamido)-3-((*S*)-2-oxopyrrolidin-3-yl)propanoate (0.2 g, 0.5 mmol) in dry THF, $NaBH_4$ (0.18 g, 10 mmol) was added portionwise at 0 °C, and CH_3OH (2 mL) was added dropwise. The reaction mixture was stirred at room temperature for 3 hours. The completion of the reaction was confirmed by TLC, and then the reaction was quenched with a saturated NH_4Cl solution (20 mL). The reaction mixture was extracted with Ethyl acetate (50 mL × 3), and the organic layers were washed with saturated NH_4Cl solution (50 mL × 3) and brine (50 mL × 3). The organic phase was dried over Na_2SO_4 and concentrated under reduced pressure to afford the white solid, *N*-((*S*)-1-hydroxy-3-((*S*)-2-oxopyrrolidin-3-yl)propan-2-yl)-2-(methyl(phenyl)amino)thiazole-4-carboxamide, with a yield of 0.16 g (90%).

To a solution of *N*-((*S*)-1-hydroxy-3-((*S*)-2-oxopyrrolidin-3-yl)propan-2-yl)-2-(methyl(phenyl)amino)thiazole-4-carboxamide (0.1 g, 0.25 mmol) n Dichloromethane, Dess-Martin periodinane (DMP, 0.11 g, 0.25 mmol) was added slowly, and the reaction mixture was stirred at room temperature. When the reactant was consumed, as confirmed by TLC, the reaction was quenched with a saturated $Na_2S_2O_3$ solution (50 mL × 3), saturated $NaHCO_3$ solution (50 mL × 3), and brine (50 mL × 3). The organic phase was dried over Na_2SO_4 and concentrated, and the residue was purified by flash

column chromatography (Dichloromethane: CH_3OH, 20:1 v/v) to afford the pure product 2-(methyl(phenyl)amino)-*N*-((*S*)-1-oxo-3-((*S*)-2-oxopyrrolidin-3-yl)propan-2-yl)thiazole-4-carboxamide as white solid, with a yield of 0.07 g (0.19 mmol, 76%). ^{1}H NMR (600 MHz, DMSO-d_6) δ 9.65 - 9.27 (m, 1H), 8.89 - 8.19 (m, 1H), 8.15 - 7.66 (m, 1H), 7.53 - 7.44 (m, 4H), 7.40 - 7.27 (m, 2H), 4.53 - 4.04 (m, 1H), 3.61 - 3.47 (m, 3H), 3.31 - 3.06 (m, 2H), 2.38 - 2.10 (m, 2H), 2.08 - 1.77 (m, 1H), 1.74 - 1.54 (m, 1H), 1.53 - 1.29 (m, 1H), 1.27 - 0.79 (m, 1H). HRMS (ESI) *m*/z 411.0481[M + K]$^+$.

Procedures for the chemical synthesis of AD02. *Tert-butyl (4-(((S)-4-methyl-1-oxo-1-(((S)-1-oxo-3-((S)-2-oxopyrrolidin-3-yl)propan-2-yl)amino)pentan-2-yl)carbamoyl) thiazol-2-yl)carbamate (AD02).* To a solution of 2-((*tert*-butoxycarbonyl)amino)thiazole-4-carboxylic acid (0.24 g, 1.0 mmol) in *N,N*-Dimethylformamide (DMF) (10 mL) at 0 °C, methyl *L*-leucinate (0.14 g, 1.0 mmol), *N,N*-Diisopropylethylamine (DIPEA, 0.129 g, 1.0 mmol), and 2-(7-Azabenzotriazol-1-yl)-*N,N,N',N'*-tetramethyluronium hexafluorophosphate (HATU, 0.38 g, 1.0 mmol) were added. The resulting solution was stirred overnight at room temperature. Then, the reaction mixture was quenched with water, and the resulting solid was collected by filtration to obtain a yellow crude solid, methyl (2-((*tert*-butoxycarbonyl)amino)thiazole-4-carbonyl)-*L*-leucinate, with a yield of 0.25 g (70%).

Methyl (2-((*tert*-butoxycarbonyl)amino)thiazole-4-carbonyl)-*L*-leucinate (0.19 g, 0.5 mmol) was added to a mixture of THF and 0.8 M aqueous LiOH (v/v = 1:3, 10 mL) at

0 °C and then warmed to room temperature. After stirring for 2 hours, the reaction solution was cooled to 0 °C, and the pH was adjusted to approximately 4 with 1 N HCl. The crude product was collected and washed with water to afford the pale-yellow solid, (2-((*tert*-butoxycarbonyl)amino)thiazole-4-carbonyl)-*L*-leucine, with a yield of 0.14 g (80%).

Methyl (*S*)-2-((*tert*-butoxycarbonyl)amino)-3-((*S*)-2-oxopyrrolidin-3-yl)propanoate (1.0 g. 3.5 mmol) was dissolved in 10 mL of Dichloromethane, and then HCl (9 mL, 4 M in dioxane) was added. The reaction mixture was stirred at ambient temperature for 12 hours and concentrated under reduced pressure to obtain a white solid, methyl (*S*)-2-amino-3-((*S*)-2-oxopyrrolidin-3-yl)propanoate, which could be used for the following step without purification.

To a solution of (2-((*tert*-butoxycarbonyl)amino)thiazole-4-carbonyl)-*L*-leucine (0.35 g, 1 mmol) in acetonitrile, *N,N,N',N'*-tetramethylchloroformamidinium hexa-fluorophosphate (TCFH, 0.27 g, 1 mmol), *N*-methylimidazole (NMI, 0.32 g, 4 mmol), and Methyl (*S*)-2-amino-3-((*S*)-2-oxopyrrolidin-3-yl)propanoate (0.18 g, 1 mmol) were added, followed by stirring at room temperature. After stirring overnight, the reaction solution was cooled to room temperature, and a solid formed. The solution was washed with a saturated aqueous NaCl solution and extracted with Ethyl acetate (10 mL × 3). The organic phase was dried with sodium sulfate and concentrated under reduced pressure to afford the pale-yellow solid, methyl (*S*)-2-((*S*)-2-(2-((*tert*-

butoxycarbonyl)amino)thiazole-4-carboxamido)-4-methylpentanamido)-3-((*S*)-2-oxo-pyrrolidin-3-yl)propanoate, with a yield of 0.36 g, (0.7 mmol, 70%).

To a solution of methyl (*S*)-2-((*S*)-2-(2-((*tert*-butoxycarbonyl)amino)thiazole-4-carboxamido)-4-methylpentanamido)-3-((*S*)-2-oxopyrrolidin-3-yl)propanoate (0.26 g, 0.5 mmol) in dry THF, $NaBH_4$ (0.18 g, 10 mmol) was added portionwise at 0 °C, and CH_3OH (2 mL) was added dropwise. The reaction mixture was stirred at room temperature for 3 hours. The completion of the reaction was confirmed by TLC, and then the reaction was quenched with a saturated NH_4Cl solution (20 mL). The reaction mixture was extracted with Ethyl acetate (50 mL × 3), and the organic layers were washed with saturated NH_4Cl solution (50 mL × 3) and brine (50 mL × 3). The organic phase was dried over Na_2SO_4 and concentrated under reduced pressure to afford the white solid, *tert*-butyl (4-(((*S*)-1-(((*S*)-1-hydroxy-3-((*S*)-2-oxopyrrolidin-3-yl)propan-2-yl)amino)-4-methyl-1-oxopentan-2-yl)carbamoyl)thiazol-2-yl)carbamate, with a yield of 0.22 g (90%).

To a solution of *tert*-butyl (4-(((*S*)-1-(((S)-1-hydroxy-3-((*S*)-2-oxopyrrolidin-3-yl)propan-2-yl)amino)-4-methyl-1-oxopentan-2-yl)carbamoyl)thiazol-2-yl)carbamate (0.13 g, 0.25 mmol) in Dichloromethane, Dess-Martin periodinane (DMP, 0.11 g, 0.25 mmol) was added slowly, and the reaction mixture was stirred at room temperature. When the reactant was consumed, as confirmed by TLC, the reaction was quenched with a saturated $Na_2S_2O_3$ solution (50 mL × 3), saturated $NaHCO_3$ solution (50 mL ×

3), and brine (50 mL × 3). The organic phase was dried over Na_2SO_4 and concentrated, and the residue was purified by flash column chromatography (Dichloromethane: Methanol, 20:1 v/v) to afford the white pure solid product, *tert*-butyl (4-(((*S*)-4-methyl-1-oxo-1-(((*S*)-1-oxo-3-((*S*)-2-oxopyrrolidin-3-yl)propan-2-yl)amino)pentan-2-yl)-carbamoyl)thiazol-2-yl)carbamate, with a yield of 0.09 g, (0.18 mmol, 70%). ^{1}H NMR (600 MHz, DMSO-d_6) δ 11.69 (s, 1H), 7.77 (s, 1H), 7.68 - 7.60 (m, 1H), 4.79 - 4.46 (m, 1H), 3.20 - 3.06 (m, 2H), 2.32 - 2.15 (m, 1H), 2.12 - 1.96 (m, 1H), 1.92 - 1.74 (m, 1H), 1.69 - 1.55 (m, 2H), 1.54 - 1.50 (m, 2H), 1.49 - 1.46 (m, 9H), 1.40 - 1.30 (m, 1H), 1.26 - 1.18 (m, 2H), 0.94 - 0.88 (m, 6H), 0.85 - 0.73 (m, 2H). HRMS (ESI) *m*/z 496.2280 $[M + H]^+$.

Procedures for the chemical synthesis of AD04. *(S)-4-methyl-N-((S)-1-oxo-3-((S)-2-oxopyrrolidin-3-yl)propan-2-yl)-2-((S)-3-phenyl-2-(2-(thiophen-2-yl)acetamido)propanamido)pentanamide (AD04).* To a solution of 2-(thiophen-2-yl)acetic acid (0.28 g, 2.0 mmol) in *N,N*-Dimethylformamide (DMF) (10 mL) at 0 °C, methyl *L*-phenylalaninate (0.36 g, 2.0 mmol), *N,N*-Diisopropylethylamine (DIPEA, 0.25 g, 2.0 mmol), and 2-(7-Azabenzotriazol-1-yl)-*N,N,N',N'*-tetramethyluronium hexafluorophosphate (HATU, 0.76 g, 2.0 mmol) were added. The resulting solution was stirred overnight at room temperature. Then, the reaction mixture was quenched with water, and the resulting solid was collected by filtration to obtain a yellow crude solid, methyl (2-(thiophen-2-yl)acetyl)-*L*-phenylalaninate, with a yield of 0.24 g (70%).

Methyl (2-(thiophen-2-yl)acetyl)-*L*-phenylalaninate (0.2 g, 1.2 mmol) was added to a mixture of THF and 0.8 M aqueous LiOH (v/v = 1:3, 10 mL) at 0 °C and then warmed to room temperature. After stirring for 2 hours, the reaction solution was cooled to 0 °C, and the pH was adjusted to about 4 with 1 N HCl. The crude product was collected and washed with water to afford the pale-yellow solid, (2-(thiophen-2-yl)acetyl)-*L*-phenylalanine, with a yield of 0.15 g (80%).

To a solution of (2-(thiophen-2-yl)acetyl)-*L*-phenylalanine (0.29 g, 1 mmol) in *N,N*-Dimethylformamide (DMF) (10 mL) at 0 °C, methyl L-leucine (0.14 g, 1.0 mmol), *N,N*-Diisopropylethylamine (DIPEA, 0.129 g, 1.0 mmol), and 2-(7-Azabenzotriazol-1-yl)-*N,N,N',N'*-tetramethyluronium hexafluorophosphate (HATU, 0.38 g, 1.0 mmol) were added. The resulting solution was stirred overnight at room temperature. Then, the reaction mixture was quenched with water, and the resulting solid was collected by filtration to obtain a yellow crude solid, methyl (2-(thiophen-2-yl)acetyl)-*L*-phenylalanyl-*L*-leucinate, with a yield of 0.32 g (70%).

Methyl (2-(thiophen-2-yl)acetyl)-*L*-phenylalanyl-*L*-leucinate (0.21 g, 0.5 mmol) was added to a mixture of THF and 0.8 M aqueous LiOH (v/v = 1:3, 10 mL) at 0 °C and then warmed to room temperature. After stirring for 2 hours, the reaction solution was cooled to 0 °C, and the pH was adjusted to about 4 with 1 N HCl. The crude product was collected and washed with water to afford the pale-yellow solid, (2-(thiophen-2-yl)acetyl)-*L*-phenylalanyl-*L*-leucine, with a yield of 0.17 g (80%).

Methyl (*S*)-2-((*tert*-butoxycarbonyl)amino)-3-((*S*)-2-oxopyrrolidin-3-yl)propanoate (1.0 g. 3.5 mmol) was dissolved in 10 mL of Dichloromethane, and then HCl (9 mL, 4 M in dioxane) was added. The reaction mixture was stirred at ambient temperature for 12 hours and concentrated under reduced pressure to obtain a white solid, methyl (*S*)-2-amino-3-((*S*)-2-oxopyrrolidin-3-yl)propanoate, which could be used for the following step without further purification.

To a solution of (2-(thiophen-2-yl)acetyl)-*L*-phenylalanyl-*L*-leucine (0.2 g, 0.5 mmol) in acetonitrile, *N,N,N',N'*-tetramethylchloroformamidinium hexafluorophosphate (TCFH, 0.14 g, 0.5 mmol), *N*-methylimidazole (NMI, 0.16 g, 2 mmol), and Methyl (*S*)-2-amino-3-((*S*)-2-oxopyrrolidin-3-yl)propanoate (0.09 g, 0.5 mmol) were added, followed by stirring at room temperature. After stirring overnight, the reaction solution was cooled to room temperature, and a solid formed. The solution was washed with a saturated aqueous NaCl solution and extracted with Ethyl acetate (10 mL × 3). The organic phase was dried with sodium sulfate and concentrated under reduced pressure to afford the pale-yellow solid, methyl (*S*)-2-((*S*)-4-methyl-2-((*S*)-3-phenyl-2-(2-(thiophen-2-yl)acetamido)propanamido)pentanamido)-3-((*S*)-2-oxopyrrolidin-3-yl)propanoate, with a yield of 0.21 g (70%).

To a solution of methyl (*S*)-2-((*S*)-4-methyl-2-((*S*)-3-phenyl-2-(2-(thiophen-2-yl)acetamido)propanamido)pentanamido)-3-((*S*)-2-oxopyrrolidin-3-yl)propanoate (0.2

g, 0.5 mmol) in dry THF, $NaBH_4$ (0.18 g, 10 mmol) was added portionwise at 0 °C, and CH_3OH (2 mL) was added dropwise. The reaction mixture was stirred at room temperature for 3 hours. The completion of the reaction was confirmed by TLC, and then the reaction was quenched with a saturated NH_4Cl solution (20 mL). The reaction mixture was extracted with Ethyl acetate (50 mL × 3), and the organic layers were washed with saturated NH_4Cl solution (50 mL × 3) and brine (50 mL × 3). The organic phase was dried over Na_2SO_4 and concentrated under reduced pressure to afford the crude (*S*)-*N*-((*S*)-1-hydroxy-3-((*S*)-2-oxopyrrolidin-3-yl)propan-2-yl)-4-methyl-2-((*S*)-3-phenyl-2-(2-(thiophen-2-yl)acetamido)propanamido)pentanamide, with a yield of 0.24 g (90%).

To a solution of (*S*)-*N*-((*S*)-1-hydroxy-3-((*S*)-2-oxopyrrolidin-3-yl)propan-2-yl)-3-phenyl-2-(2-(thiophen-2-yl)acetamido)propanamide (0.13 g, 0.25 mmol) in Dichloromethane, Dess-Martin periodinane (DMP, 0.11 g, 0.25 mmol) was added slowly, and the reaction mixture was stirred at room temperature. When the reactant was consumed, as confirmed by TLC, the reaction was quenched with a saturated $Na_2S_2O_3$ solution (50 mL × 3), saturated $NaHCO_3$ solution (50 mL × 3), and brine (50 mL × 3). The organic phase was dried over Na_2SO_4 and concentrated, then the residue was purified by flash column chromatography (Dichloromethane: CH_3OH, 20:1 v/v) to afford the white pure solid product, (*S*)-4-methyl-*N*-((*S*)-1-oxo-3-((*S*)-2-oxopyrrolidin-3-yl)propan-2-yl)-2-((*S*)-3-phenyl-2-(2-(thiophen-2-yl)acetamido)propanamido)pentanamide, with a yield of 0.09 g (0.17 mmol, 70%). ^1H NMR (600 MHz, DMSO-d_6) δ

13.15 (s, 1H), 8.57 - 8.10 (m, 1H), 8.04 - 7.69 (m, 3H), 7.68 - 7.40 (m, 2H), 7.39 - 7.26 (m, 1H), 7.26 - 7.22 (m, 2H), 7.22 - 7.14 (m, 2H), 6.95 - 6.70 (m, 1H), 5.05 - 4.34 (m, 1H), 4.32 - 3.81 (m, 1H), 3.78 - 3.46 (m, 1H), 3.25 - 2.87 (m, 2H), 2.86 - 2.54 (m, 1H), 2.39 - 2.02 (m, 1H), 2.01 - 1.66 (m, 1H), 1.65 - 1.29 (m, 3H), 1.27 - 0.94 (m, 2H), 0.87 - 0.44 (m, 6H). HRMS (ESI) *m*/z 541.2488 $[M + H]^+$.

Procedures for the chemical synthesis of AD05. *2-Chloro-N-((S)-4-methyl-1-oxo-1-(((S)-1-oxo-3-((S)-2-oxopyrrolidin-3-yl)propan-2-yl)amino)pentan-2-yl)thiazole-4-carboxamide (AD05).* To a solution of 2-chlorothiazole-4-carboxylic acid (0.16 g, 1 mmol) in *N,N*-Dimethylformamide (DMF) (10 mL) at 0 °C, methyl *L*-leucine (0.14 g, 1.0 mmol), *N,N*-Diisopropylethylamine (DIPEA, 0.129 g, 1.0 mmol), and 2-(7-Azabenzotriazol-1-yl)-*N,N,N',N'*-tetramethyluronium hexafluorophosphate (HATU, 0.38 g, 1.0 mmol) were added. The resulting solution was stirred overnight at room temperature. Then, the reaction mixture was quenched with water, and the resulting solid was collected by filtration to obtain a yellow crude solid, methyl (2-chlorothiazole-4-carbonyl)-*L*-leucinate, with a yield of 0.19 g (70%).

Methyl (2-chlorothiazole-4-carbonyl)- *L*-leucinate (0.15 g, 0.5 mmol) was added to a mixture of THF and 0.8 M aqueous LiOH (v/v = 1:3, 10 mL) at 0 °C and then warmed to room temperature. After stirring for 2 hours, the reaction solution was cooled to 0 °C, and the pH was adjusted to about 4 with 1 N HCl. The crude product was collected and washed with water to afford the pale-yellow solid, (2-chlorothiazole-4-carbonyl)-

L-leucine, with a yield of 0.22 g (80%).

Methyl(*S*)-2-((*tert*-butoxycarbonyl)amino)-3-((*S*)-2-oxopyrrolidin-3-yl)propanoate (1.0 g. 3.5 mmol) was dissolved in 10 mL of Dichloromethane, and then HCl (9 mL, 4 M in dioxane) was added. The reaction mixture was stirred at ambient temperature for 12 hours and concentrated under reduced pressure to obtain a white solid, methyl (*S*)-2-amino-3-((*S*)-2-oxopyrrolidin-3-yl)propanoate, which could be used for the following step without further purification.

To a solution of (2-chlorothiazole-4-carbonyl)-*L*-leucine (0.14 g, 0.5 mmol) in acetonitrile, *N,N,N',N'*-tetramethylchloroformamidinium hexafluorophosphate (TCFH, 0.14 g, 0.5 mmol), *N*-methylimidazole (NMI, 0.16 g, 2 mmol), and Methyl (*S*)-2-amino-3-((*S*)-2-oxopyrrolidin-3-yl)propanoate (0.09 g, 0.5 mmol) were added, followed by stirring at room temperature. After stirring overnight, the reaction solution was cooled to room temperature, and a solid formed. The solution was washed with a saturated aqueous NaCl solution and extracted with Ethyl acetate (10 mL × 3). The organic phase was dried with sodium sulfate and concentrated under reduced pressure to afford the pale-yellow solid, methyl (*S*)-2-((*S*)-2-(2-chlorothiazole-4-carboxamido)-4-methyl-pentanamido)-3-((*S*)-2-oxopyrrolidin-3-yl)propanoate, with a yield of 0.31 g (70%).

To a solution of methyl (*S*)-2-((*S*)-2-(2-chlorothiazole-4-carboxamido)-4-methyl-pentanamido)-3-((*S*)-2-oxopyrrolidin-3-yl)propanoate (0.22 g, 0.5 mmol) in dry THF,

$NaBH_4$ (0.18 g, 10 mmol) was added portionwise at 0 °C, and CH_3OH (2 mL) was added dropwise. The reaction mixture was stirred at room temperature for 3 hours. The completion of the reaction was confirmed by TLC, and then the reaction was quenched with a saturated NH_4Cl solution (20 mL). The reaction mixture was extracted with Ethyl acetate (50 mL × 3), and the organic layers were washed with saturated NH_4Cl solution (50 mL × 3) and brine (50 mL × 3). The organic phase was dried over Na_2SO_4 and concentrated under reduced pressure to afford the crude 2-chloro-*N*-((*S*)-1-(((*S*)-1-hydroxy-3-((*S*)-2-oxopyrrolidin-3-yl)propan-2-yl)amino)-4-methyl-1-oxo-pentan-2-yl)thiazole-4-carboxamide, with a yield of 0.37 g (90%).

To a solution of 2-chloro-*N*-((*S*)-1-(((*S*)-1-hydroxy-3-((*S*)-2-oxopyrrolidin-3-yl) propan-2-yl)amino)-4-methyl-1-oxopentan-2-yl)thiazole-4-carboxamide, (0.11 g, 0.25 mmol) in Dichloromethane, Dess-Martin periodinane (DMP, 0.11 g, 0.25 mmol) was added slowly, and the reaction mixture was stirred at room temperature. When the reactant was consumed, as confirmed by TLC, the reaction was quenched with a saturated $Na_2S_2O_3$ solution (50 mL × 3), saturated $NaHCO_3$ solution (50 mL × 3), and brine (50 mL × 3). The organic phase was dried over Na_2SO_4 and concentrated, then the residue was purified by flash column chromatography (Dichloromethane: CH_3OH, 20:1 v/v) to afford the white pure solid product, *2-chloro-N-((S)-4-methyl-1-oxo-1-(((S)-1-oxo-3-((S)-2-oxopyrrolidin-3-yl)propan-2-yl)amino)pentan-2-yl)thiazole-4-carboxamide,* with a yield of 0.14 g (0.17 mmol, 70%). ^1H NMR (600 MHz, DMSO-d_6) δ 8.35 - 8.24 (m, 1H), 8.23 - 8.02 (m, 1H), 7.77 - 7.39 (m, 1H), 5.77 (s, 1H), 4.82 -

4.11 (m, 2H), 3.20 - 2.91 (m, 2H), 2.34 - 1.95 (m, 2H), 1.94 - 1.69 (m, 1H), 1.68 - 1.60 (m, 1H), 1.58 - 1.44 (m, 2H), 1.39 - 1.17 (m, 2H), 0.90 - 0.84 (m, 6H). HRMS (ESI) *m*/z 415.1203 $[M + H]^+$; 437.102 $[M + Na]^+$, 453.0763 $[M + K]^+$

Procedures for the chemical synthesis of AD06. *2-Chloro-N-((S)-3-methyl-1-(((S)-4-methyl-1-oxo-1-(((S)-1-oxo-3-((S)-2-oxopyrolidin- 3-yl)propan-2-yl)amino)pentan-2-yl)amino)-1-oxobutan-2-yl)thiazole-4-carboxamide (AD06).* To a solution of 2-chlorothiazole-4-carboxylic acid (0.33 g, 2.0 mmol) in *N,N*-Dimethylformamide (DMF) (10 mL) at 0 °C, methyl *L*-valinate (0.36 g, 2.0 mmol), *N,N*-Diisopropylethylamine (DIPEA, 0.25 g, 2.0 mmol), and 2-(7-Azabenzotriazol-1-yl)-*N,N,N',N'*-tetramethyluronium hexafluorophosphate (HATU, 0.76 g, 2.0 mmol) were added. The resulting solution was stirred overnight at room temperature. Then, the reaction mixture was quenched with water, and the resulting solid was collected by filtration to obtain a yellow crude solid, methyl (2-chlorothiazole-4-carbonyl)-*L*-valinate, with a yield of 0.38 g (70%).

Methyl (2-chlorothiazole-4-carbonyl)-*L*-valinate (0.27 g, 1.0 mmol) was added to a mixture of THF and 0.8 M aqueous LiOH (v/v = 1:3, 10 mL) at 0 °C and then warmed to room temperature. After stirring for 2 hours, the reaction solution was cooled to 0 °C, and the pH was adjusted to about 4 with 1 N HCl. The crude product was collected and washed with water to afford the pale-yellow solid, (2-chlorothiazole-4-carbonyl)-*L*-valine, with a yield of 0.21 g (80%).

To a solution of (2-chlorothiazole-4-carbonyl)-*L*-valine (0.27 g, 1 mmol) in *N,N*-Dimethylformamide (DMF) (10 mL) at 0 °C, methyl *L*-leucine (0.14 g, 1.0 mmol), *N,N*-Diisopropylethylamine (DIPEA, 0.129 g, 1.0 mmol), and 2-(7-Azabenzotriazol-1-yl)-*N,N,N',N'*-tetramethyluronium hexafluorophosphate (HATU, 0.38 g, 1.0 mmol) were added. The resulting solution was stirred overnight at room temperature. Then, the reaction mixture was quenched with water, and the resulting solid was collected by filtration to obtain a yellow crude solid, methyl (2-chlorothiazole-4-carbonyl)-*L*-valyl-*L*-leucinate, with a yield of 0.28 g (70%).

Methyl (2-chlorothiazole-4-carbonyl)-*L*-valyl-*L*-leucinate (0.18 g, 0.5 mmol) was added to a mixture of THF and 0.8 M aqueous LiOH (v/v = 1:3, 10 mL) at 0 °C and then warmed to room temperature. After stirring for 2 hours, the reaction solution was cooled to 0 °C, and the pH was adjusted to about 4 with 1 N HCl. The crude product was collected and washed with water to afford the pale-yellow solid, (2-chlorothiazole-4-carbonyl)-*L*-valyl-*L*-leucine, with a yield of 0.15 g (80%).

Methyl (*S*)-2-((*tert*-butoxycarbonyl)amino)-3-((*S*)-2-oxopyrrolidin-3-yl)propanoate (1.0 g. 3.5 mmol) was dissolved in 10 mL of Dichloromethane, and then HCl (9 mL, 4 M in dioxane) was added. The reaction mixture was stirred at ambient temperature for 12 hours and concentrated under reduced pressure to obtain a white solid, methyl (*S*)-2-amino-3-((*S*)-2-oxopyrrolidin-3-yl)propanoate, which could be used for the

following step without further purification.

To a solution of (2-chlorothiazole-4-carbonyl)-*L*-valyl-*L*-leucine (0.19 g, 0.5 mmol) in acetonitrile, *N,N,N′,N′*-tetramethylchloroformamidinium hexafluorophosphate (TCFH, 0.14 g, 0.5 mmol), *N*-methylimidazole (NMI, 0.16 g, 2 mmol), and Methyl (*S*)-2-amino-3-((*S*)-2-oxopyrrolidin-3-yl)propanoate (0.09 g, 0.5 mmol) were added, followed by stirring at room temperature. After stirring overnight, the reaction solution was cooled to room temperature, and a solid formed. The solution was washed with a saturated aqueous NaCl solution and extracted with Ethyl acetate (10 mL × 3). The organic phase was dried with sodium sulfate and concentrated under reduced pressure to afford the pale-yellow solid, methyl (*S*)-2-((*S*)-2-((*S*)-2-(2-chlorothiazole-4-carboxamido)-3-methylbutanamido)-4-methylpentanamido)-3-((*S*)-2-oxopyrrolidin-3-yl)propanoate, with a yield of 0.19 g (70%).

To a solution of methyl (*S*)-2-((*S*)-2-((*S*)-2-(2-chlorothiazole-4-carboxamido)-3-methylbutanamido)-4-methylpentanamido)-3-((*S*)-2-oxopyrrolidin-3-yl)propanoate (0.27 g, 0.5 mmol) in dry THF, $NaBH_4$ (0.18 g, 10 mmol) was added portionwise at 0 °C, and CH_3OH (2 mL) was added dropwise. The reaction mixture was stirred at room temperature for 3 hours. The completion of the reaction was confirmed by TLC, and then the reaction was quenched with a saturated NH_4Cl solution (20 mL). The reaction mixture was extracted with Ethyl acetate (50 mL × 3), and the organic layers were washed with saturated NH_4Cl solution (50 mL × 3) and brine (50 mL × 3). The organic

phase was dried over Na_2SO_4 and concentrated under reduced pressure to afford the crude (*S*)-*N*-((*S*)-1-hydroxy-3-((*S*)-2-oxopyrrolidin-3-yl)propan-2-yl)-4-methyl-2-((*S*)-3-phenyl-2-(2-(thiophen-2-yl)acetamido)propanamido)pentanamide, with a yield of 0.23 g (90%).

To a solution of 2-chloro-*N*-((*S*)-1-(((*S*)-1-(((*S*)-1-hydroxy-3-((*S*)-2-oxopyrrolidin-3-yl)propan-2-yl)amino)-4-methyl-1-oxopentan-2-yl)amino)-3-methyl-1-oxobutan-2-yl)thiazole-4-carboxamide (0.13 g, 0.25 mmol) in Dichloromethane, Dess-Martin periodinane (DMP, 0.11 g, 0.25 mmol) was added slowly, and the reaction mixture was stirred at room temperature. When the reactant was consumed, as confirmed by TLC, the reaction was quenched with a saturated $Na_2S_2O_3$ solution (50 mL × 3), saturated $NaHCO_3$ solution (50 mL × 3), and brine (50 mL × 3). The organic phase was dried over Na_2SO_4 and concentrated, then the residue was purified by flash column chromatography (Dichloromethane: CH_3OH, 20:1 v/v) to afford the white pure solid product, 2-chloro-*N*-((*S*)-3-methyl-1-(((*S*)-4-methyl-1-oxo-1-(((*S*)-1-oxo-3-((*S*)-2-oxo pyrrolidin-3-yl)propan-2-yl)amino)pentan-2-yl)amino)-1-oxobutan-2-yl)thiazole-4-carboxamide, with a yield of 0.09 g (0.17 mmol, 70%). ^1H NMR (600 MHz, DMSO-d_6) δ 8.60 - 8.39 (m, 1H), 8.35 - 8.29 (m, 1H), 8.07 - 7.95 (m, 1H), 7.68 - 7.45 (m, 1H), 6.05 - 5.57 (m, 1H), 4.80 - 4.37 (m, 2H), 4.36 - 4.10 (m, 1H), 3.18 - 2.93 (m, 2H), 2.27 - 2.15 (m, 1H), 2.12 - 2.03 (m, 2H), 1.65 - 1.53 (m, 2H), 1.52 - 1.37 (m, 3H), 1.31 - 1.17 (m, 2H), 0.91 - 0.86 (m, 6H), 0.86 - 0.81 (m, 6H). HRMS (ESI) *m*/z 514.2899 $[M + H]^+$; 536.1716 $[M + Na]^+$.

2-Chloro-N-((S)-1-(((S)-1-(((S)-1-cyano-2-((S)-2-oxopyrrolidin-3-yl)ethyl)amino)-4-methyl-1-oxopentan-2-yl)amino)-3-methyl-1-oxobutan-2-yl)thiazole-4-carboxamide (AD06CN01). (*S*)-2-Amino-3-((*S*)-2-oxopyrrolidin-3-yl)propanenitrile hydrochloride was chosen to replace methyl (*S*)-2-amino-3-((*S*)-2-oxopyrrolidin-3-yl)propanoate. AD06CN01 was prepared by following a similar procedure to that used for AD06. White solid (34 mg, 0.068 mmol, 70%, R_f = 0.33, Dichloromethane: Methanol = 20:1). ^{1}H NMR (600 MHz, DMSO-d_6) δ 8.96 - 8.78 (m, 1H), 8.61 - 8.35 (m, 1H), 8.34 - 8.27 (m, 1H), 8.08 - 7.95 (m, 1H), 7.76 - 7.70 (m, 1H), 5.00 - 4.89 (m, 1H), 4.41 - 4.34 (m, 1H), 4.34 - 4.20 (m, 1H), 3.19 - 3.04 (m, 2H), 2.39 - 2.21 (m, 1H), 2.18 - 2.08 (m, 2H), 2.07 - 2.02 (m, 1H), 1.80 - 1.63 (m, 2H), 1.55 - 1.26 (m, 3H), 0.90 - 0.83 (m, 12H). HRMS (ESI) *m*/z 511.18922 $[M + H]^+$; 533.1716 $[M + Na]^+$.

Tert-Butyl (4-(((S)-1-(((S)-1-cyano-2-((S)-2-oxopyrrolidin-3-yl)ethyl)amino)-1-oxo-3-phenylpropan-2-yl)carbamoyl)thiazol-2-yl)carbamate (CN01). (*S*)-2-Amino-3-((*S*)-2-oxopyrrolidin-3-yl)propanenitrile hydrochloride was chosen to replace methyl (*S*)-2-amino-3-((*S*)-2-oxopyrrolidin-3-yl)propanoate. *L*-Phenylalanine was chosen to replace methyl *L*-valinate. CN01 was prepared by following a similar procedure to that used for AD02. White solid (34 mg, 0.065 mmol, 70%, R_f = 0.33, Dichloromethane: Methanol = 20:1). ^{1}H NMR (600 MHz, DMSO-d_6) δ 11.74 (s, 1H), 9.11 (d, J = 7.7 Hz, 1H), 7.79 - 7.72 (m, 3H), 7.28 - 7.24 (m, 2H), 7.22 - 7.20 (m, 1H), 7.19 - 7.17 (m, 2H), 4.98 - 4.93 (m, 1H), 4.74 - 4.70 (m, 1H), 3.16 - 3.11 (m, 2H), 3.10 - 3.02 (m, 2H), 2.32

- 2.26 (m, 1H), 2.14 - 2.08 (m, 2H), 1.79 (ddd, J = 13.5, 9.1, 7.0 Hz, 1H), 1.72 - 1.66 (m, 1H), 1.48 (s, 9H). HRMS (ESI) *m*/z 527.2072 $[M + H]^+$; 549.1909 $[M + Na]^+$.

Tert-Butyl (4-(((S)-1-(((S)-1-cyano-2-((S)-2-oxopyrrolidin-3-yl)ethyl)amino)-1-oxo-3-phenylpropan-2-yl)carbamoyl)oxazol-2-yl)carbamate (CN02). 2-((*Tert*-butoxycarbonyl)amino)oxazole-4-carboxylic acid was chosen to replace 2-((*tert*-butoxycarbonyl)amino)thiazole-4-carboxylic acid. CN02 was prepared by following a similar procedure to that used for CN01. White solid (34 mg, 0.065 mmol, 70%, R_f = 0.33, Dichloromethane: Methanol = 20:1). ^{1}H NMR (600 MHz, DMSO-d_6) δ 10.88 (s, 1H), 9.03 - 9.00 (m, 1H), 8.32 (s, 1H), 7.90 - 7.87 (m, 1H), 7.73 (s, 1H), 7.27 - 7.24 (m, 2H), 7.23 - 7.22 (m, 1H), 7.22 - 7.18 (m, 2H), 4.98 - 4.94 (m, 1H), 4.71 - 4.66 (m, 1H), 3.16 - 3.11 (m, 2H), 3.10 - 3.06 (m, 2H), 2.34 - 2.29 (m, 1H), 2.15 - 2.10 (m, 2H), 1.82 - 1.76 (m, 1H), 1.73 - 1.66 (m, 1H), 1.48 - 1.45 (m, 9H). HRMS (ESI) *m*/z 511.2305 $[M + H]^+$; 533.2145 $[M + Na]^+$.

The NMR and mass spectra of the synthesized compounds are given in ^{1}H NMR and high-resolution mass spectra.

^{1}H NMR and high-resolution mass spectra

The ^{1}H NMR and high-resolution mass spectra of synthesized compounds synthesized are shown as follows.

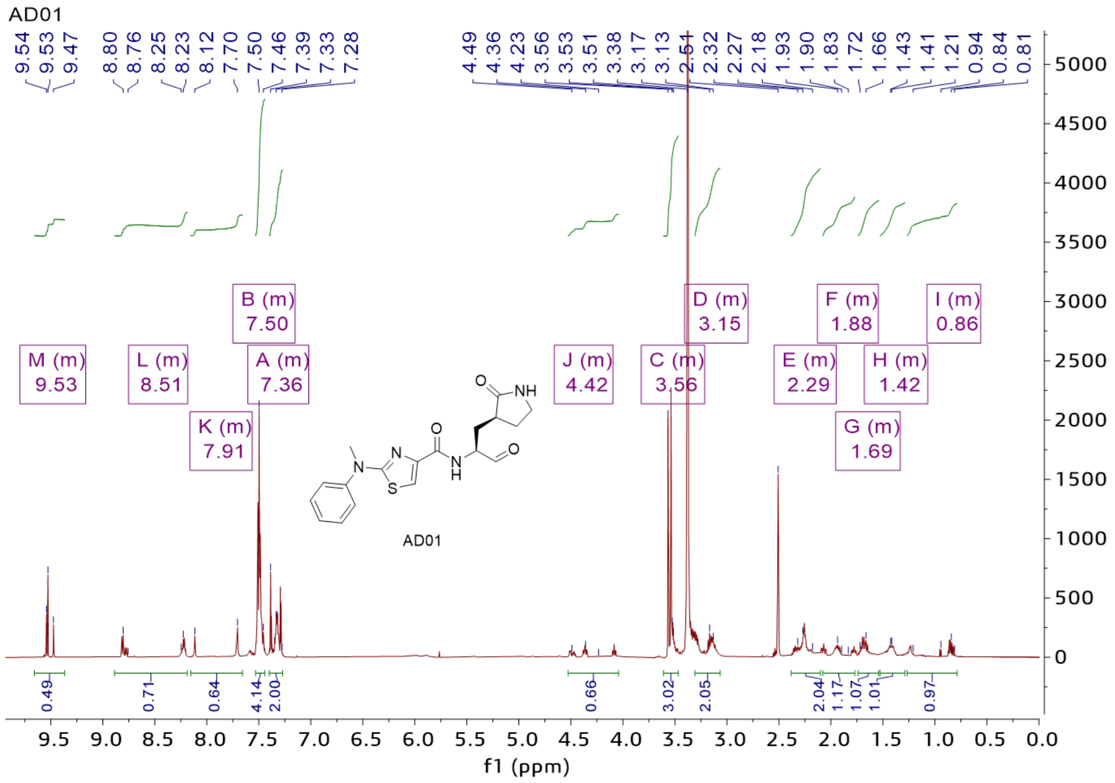
AD01
B (m) 7.50
M (m) 9.53
L (m) 8.51
A (m) 7.36
K (m) 7.91
J (m) 4.42
C (m) 3.56
D (m) 3.15
E (m) 2.29
F (m) 1.88
H (m) 1.42
G (m) 1.69
I (m) 0.86
AD01
f1 (ppm)

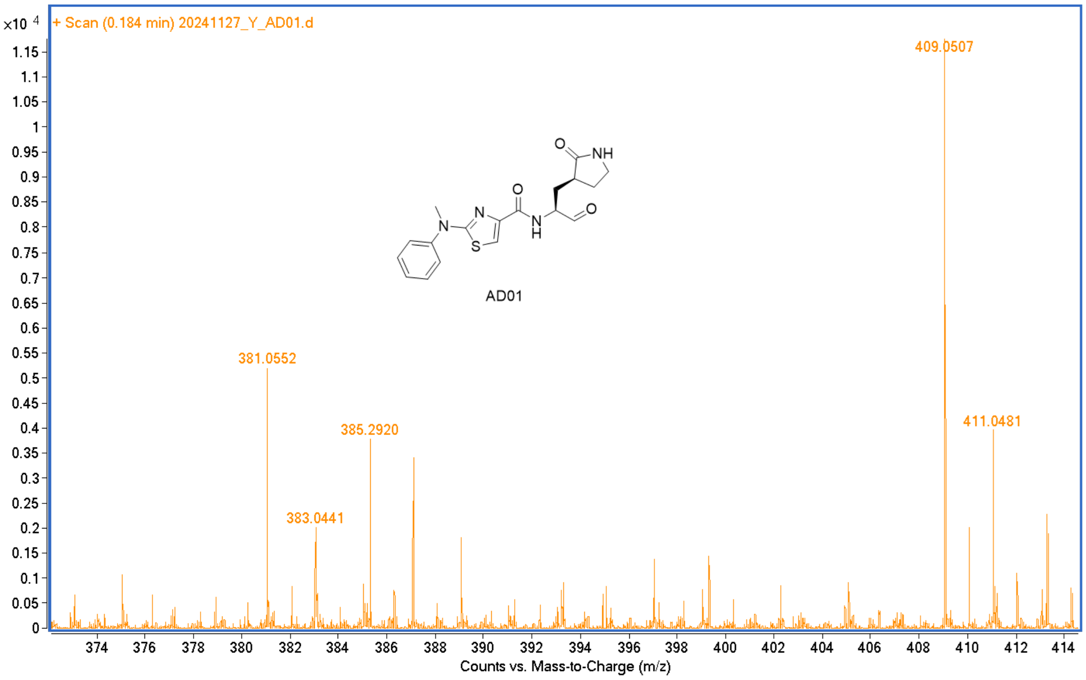
+ Scan (0.184 min) 20241127_Y_AD01.d
409.0507
381.0552
385.2920
383.0441
411.0481
AD01
Counts vs. Mass-to-Charge (m/z)

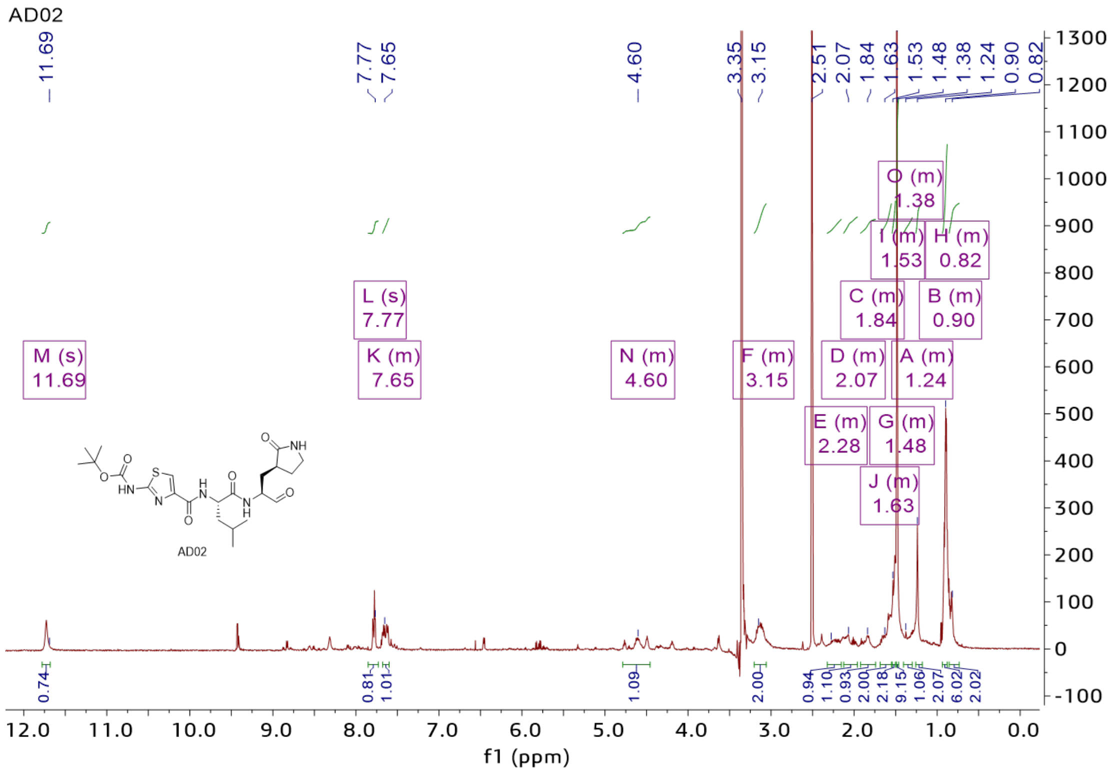
AD02
f1 (ppm)
M (s) 11.69
L (s) 7.77
K (m) 7.65
N (m) 4.60
F (m) 3.15
D (m) 2.07
E (m) 2.28
C (m) 1.84
J (m) 1.63
I (m) 1.53
G (m) 1.48
O (m) 1.38
A (m) 1.24
B (m) 0.90
H (m) 0.82

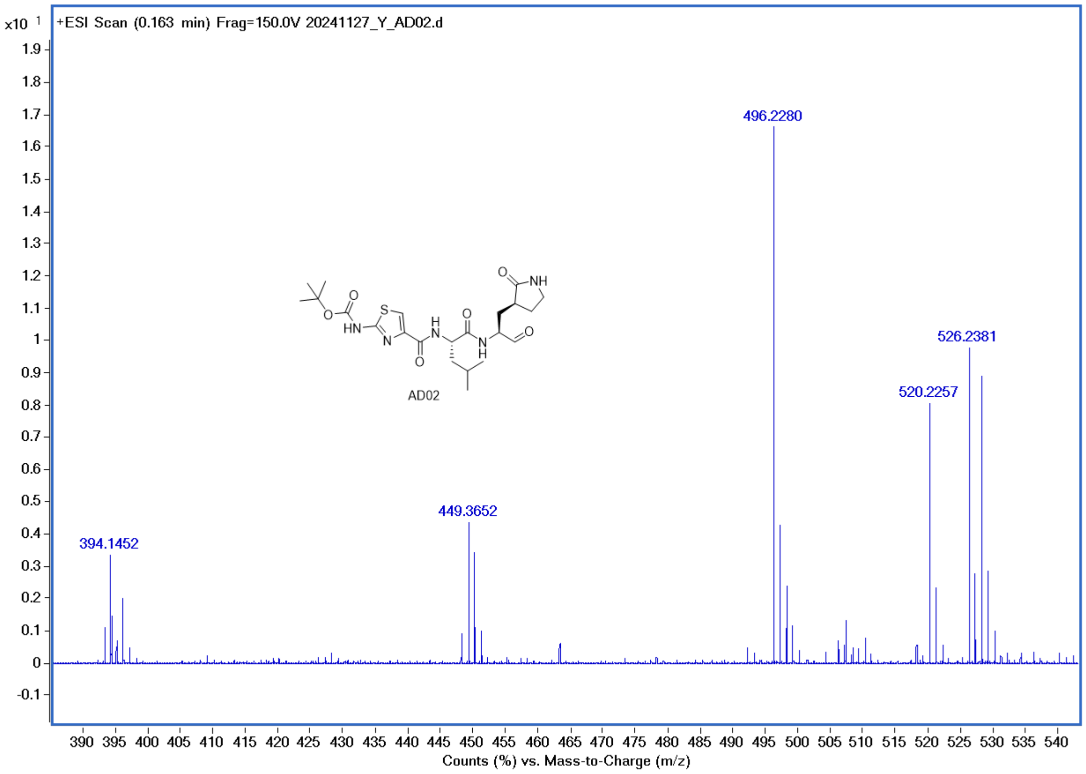
+ESI Scan (0.163 min) Frag=150.0V 20241127_Y_AD02.d
496.2280
526.2381
520.2257
449.3652
394.1452
AD02
Counts (%) vs. Mass-to-Charge (m/z)

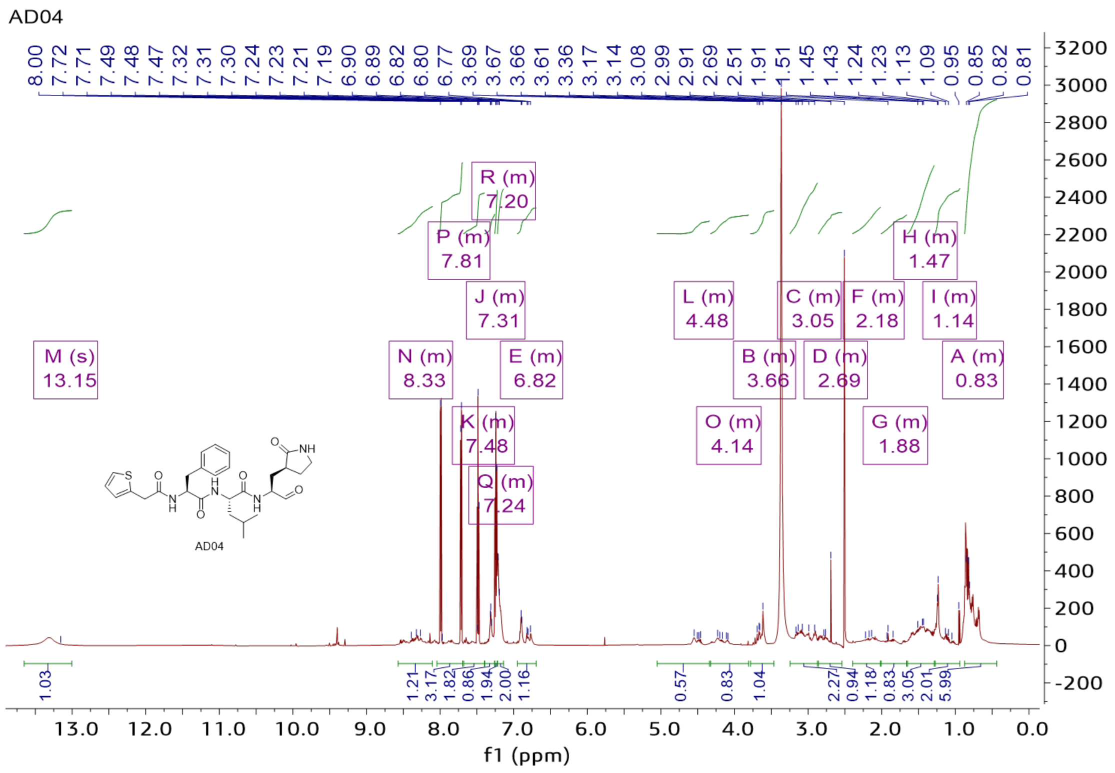
AD04
M (s) 13.15
N (m) 8.33
P (m) 7.81
J (m) 7.31
K (m) 7.48
R (m) 7.20
Q (m) 7.24
E (m) 6.82
L (m) 4.48
O (m) 4.14
B (m) 3.66
C (m) 3.05
D (m) 2.69
F (m) 2.18
G (m) 1.88
H (m) 1.47
I (m) 1.14
A (m) 0.83
f1 (ppm)

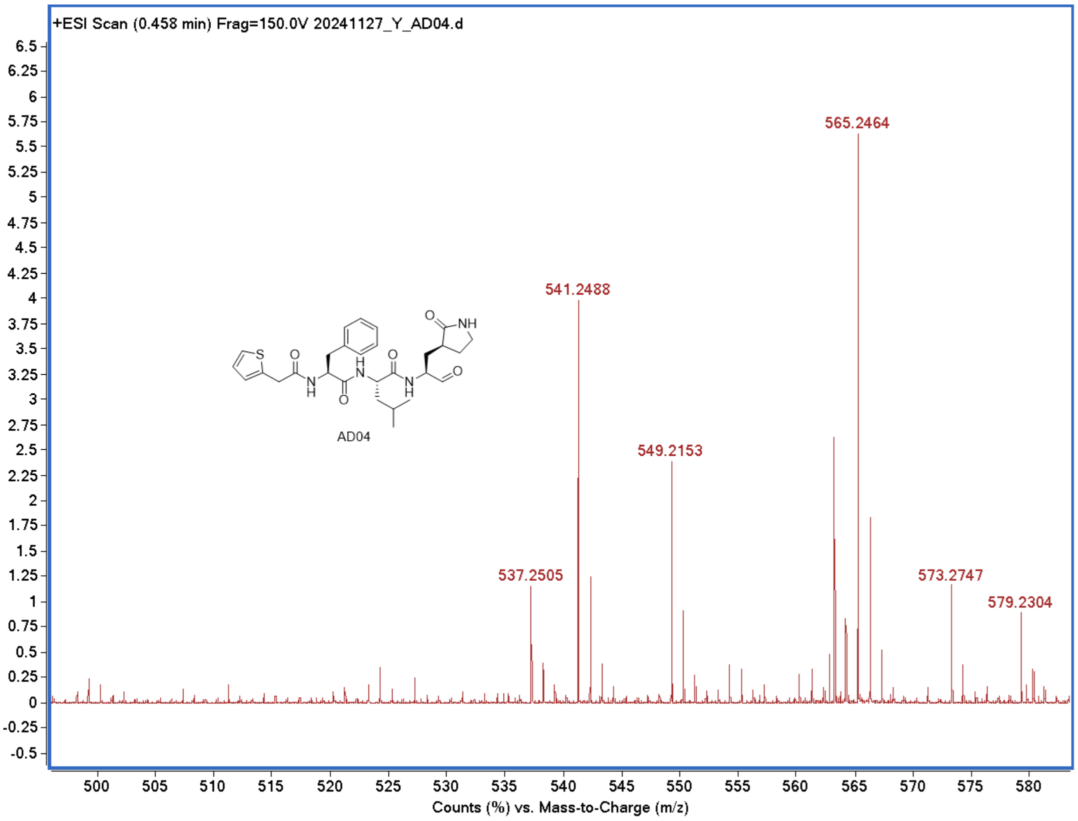
+ESI Scan (0.458 min) Frag=150.0V 20241127_Y_AD04.d
565.2464
541.2488
549.2153
537.2505
573.2747
579.2304
AD04
Counts (%) vs. Mass-to-Charge (m/z)

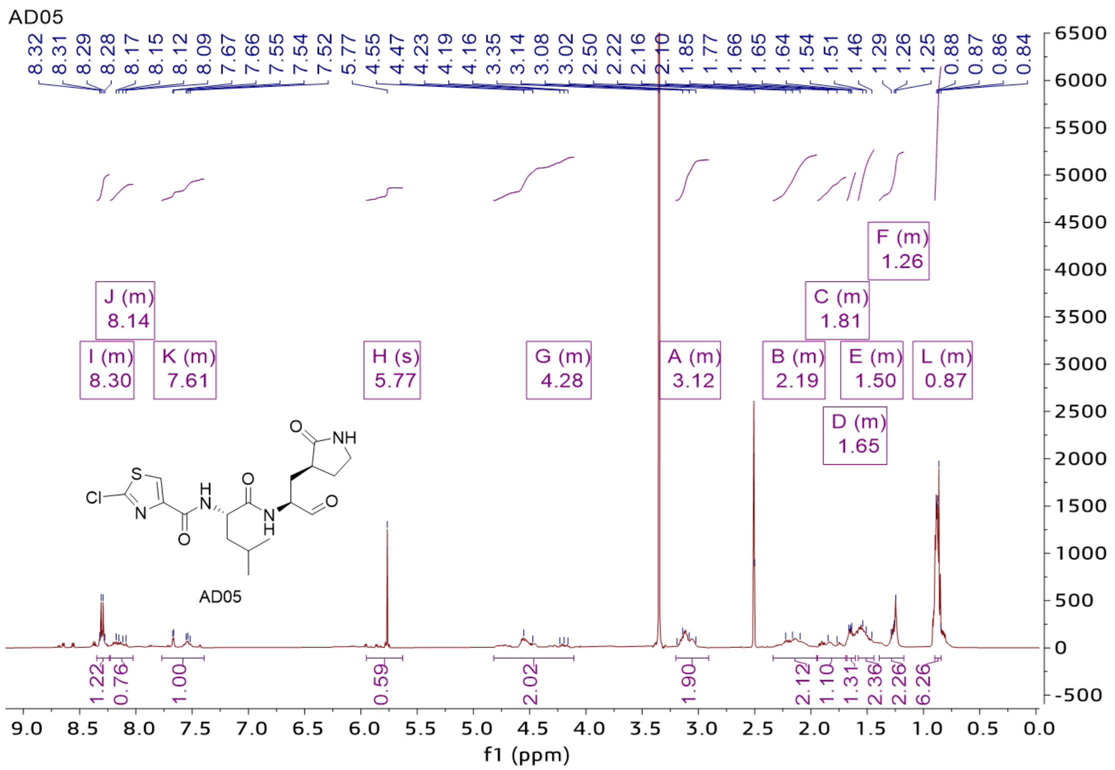
AD05
J (m) 8.14
I (m) 8.30
K (m) 7.61
H (s) 5.77
G (m) 4.28
A (m) 3.12
B (m) 2.19
C (m) 1.81
E (m) 1.50
D (m) 1.65
F (m) 1.26
L (m) 0.87
AD05
f1 (ppm)

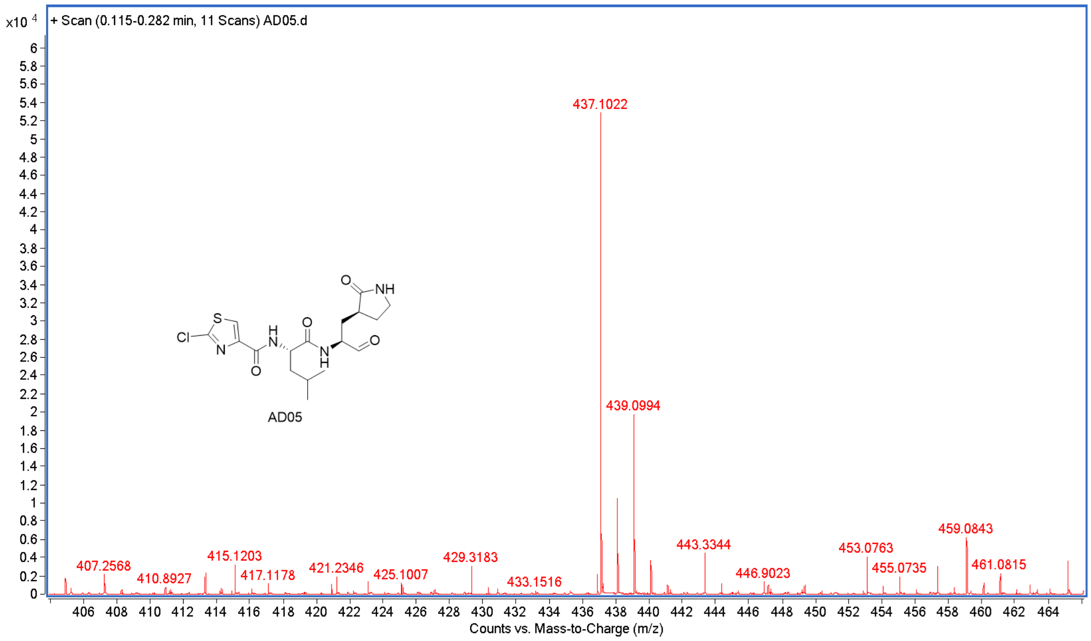
+ Scan (0.115-0.282 min, 11 Scans) AD05.d
437.1022
439.0994
AD05
Counts vs. Mass-to-Charge (m/z)

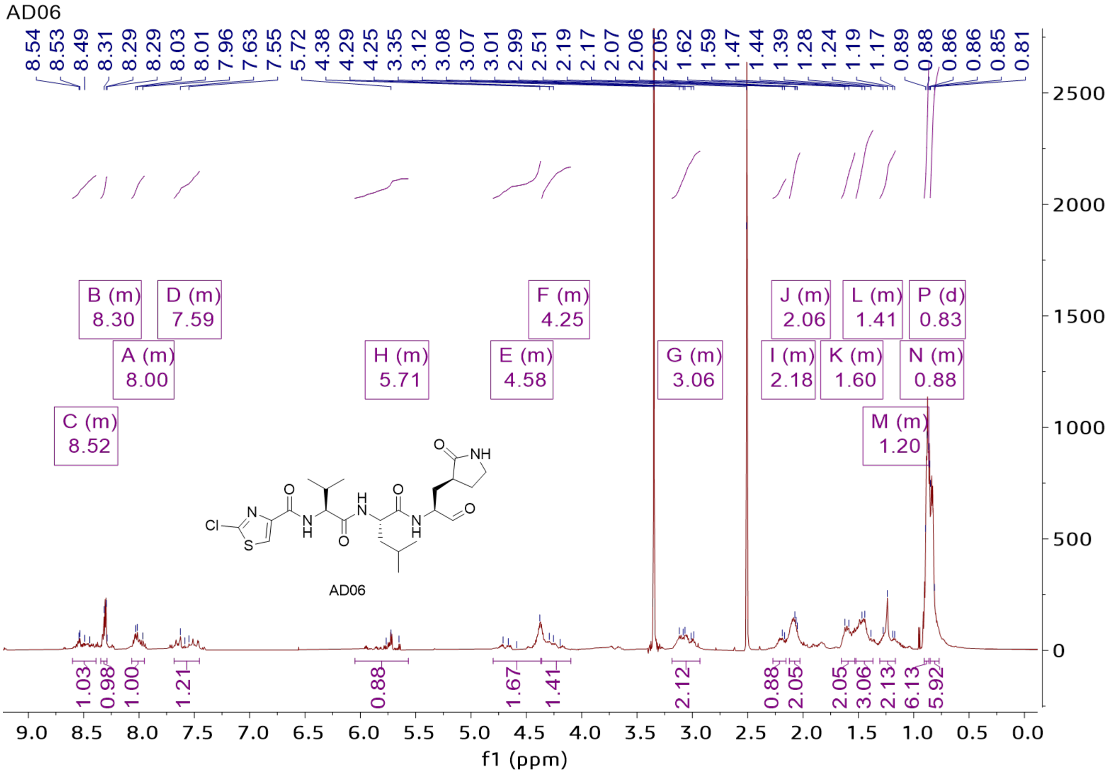
AD06
8.54 8.53 8.49 8.31 8.29 8.29 8.03 8.01 7.96 7.63 7.55 5.72 4.38 4.29 4.25 3.35 3.12 3.08 3.07 3.01 2.99 2.51 2.19 2.17 2.07 2.06 2.05 1.62 1.59 1.47 1.44 1.39 1.28 1.24 1.19 1.17 0.89 0.88 0.86 0.86 0.85 0.81
B (m) 8.30
D (m) 7.59
A (m) 8.00
C (m) 8.52
H (m) 5.71
F (m) 4.25
E (m) 4.58
G (m) 3.06
J (m) 2.06
I (m) 2.18
L (m) 1.41
K (m) 1.60
P (d) 0.83
N (m) 0.88
M (m) 1.20
AD06
1.03 0.98 1.00 1.21 0.88 1.67 1.41 2.12 0.88 2.05 2.05 3.06 2.13 6.13 5.92
f1 (ppm)

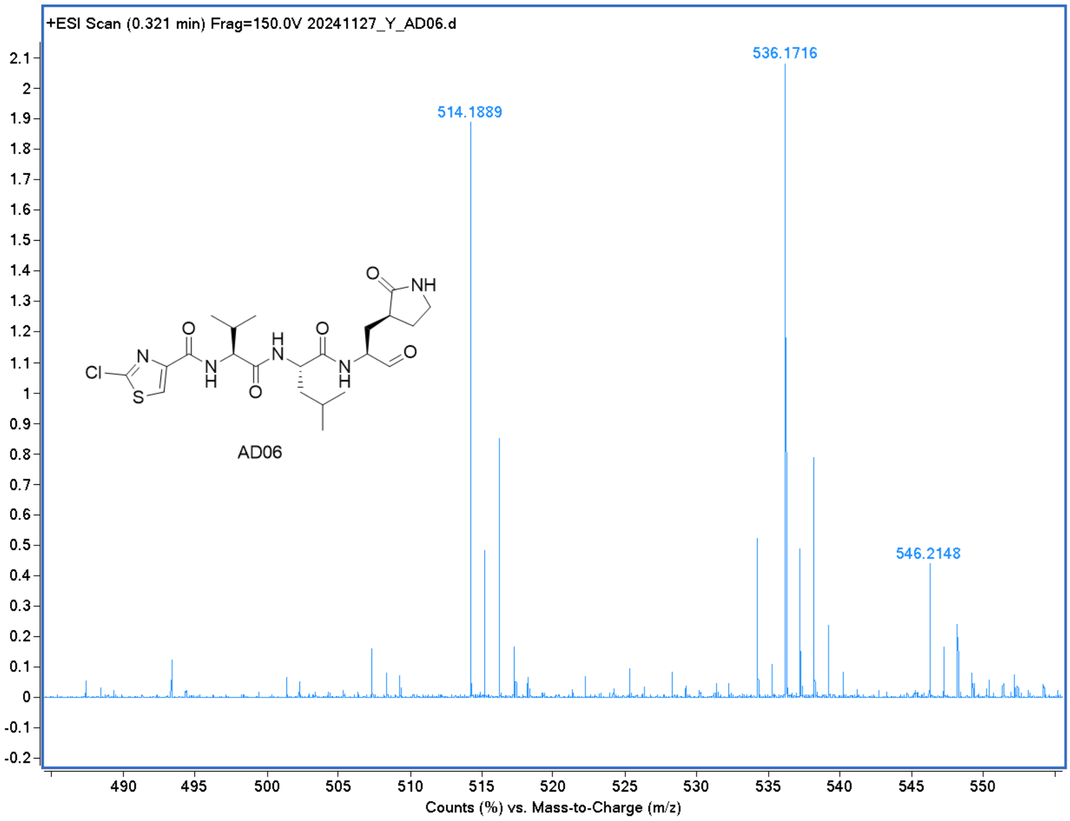
+ESI Scan (0.321 min) Frag=150.0V 20241127_Y_AD06.d
536.1716
514.1889
546.2148
AD06
Counts (%) vs. Mass-to-Charge (m/z)

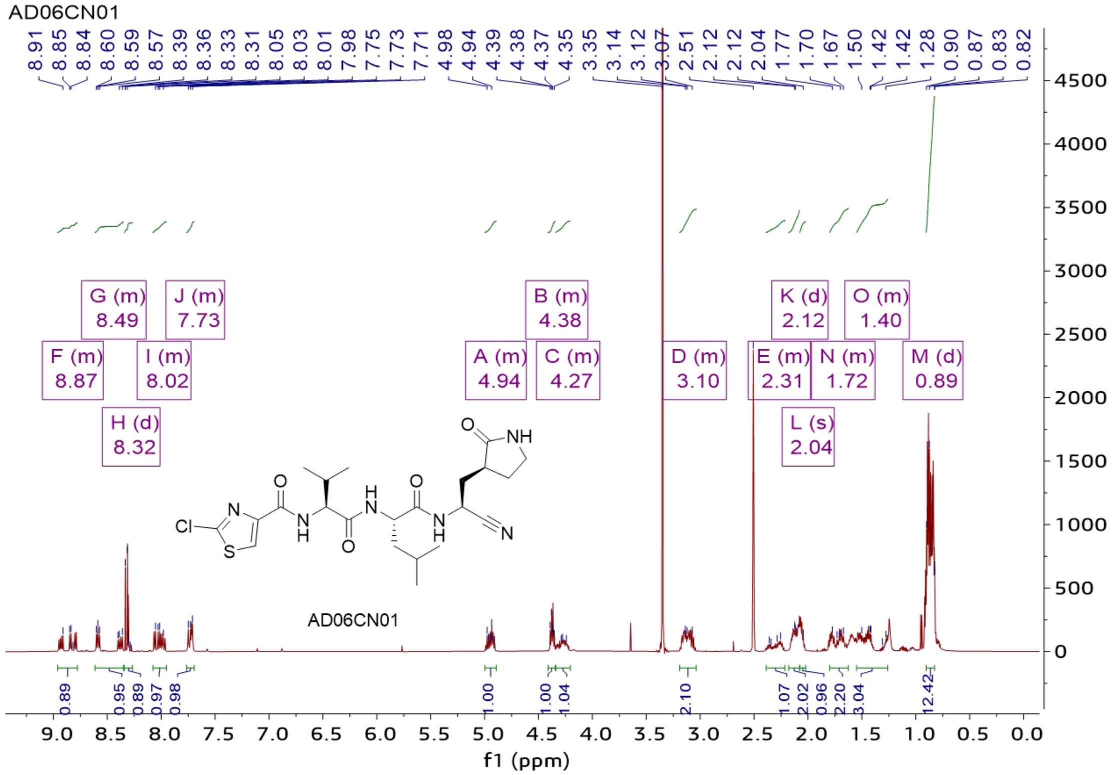
AD06CN01
F (m) 8.87
G (m) 8.49
H (d) 8.32
I (m) 8.02
J (m) 7.73
A (m) 4.94
B (m) 4.38
C (m) 4.27
D (m) 3.10
E (m) 2.31
K (d) 2.12
L (s) 2.04
N (m) 1.72
O (m) 1.40
M (d) 0.89
AD06CN01
f1 (ppm)

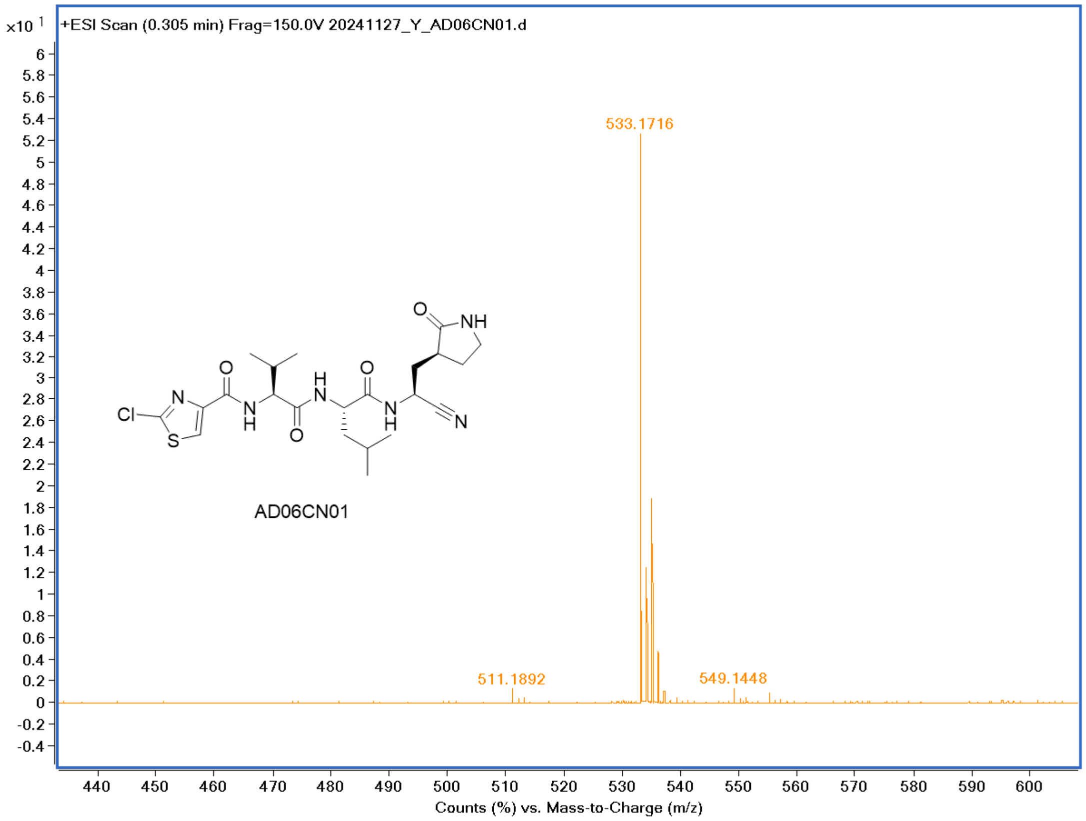
+ESI Scan (0.305 min) Frag=150.0V 20241127_Y_AD06CN01.d
533.1716
511.1892
549.1448
AD06CN01
Counts (%) vs. Mass-to-Charge (m/z)

CN01

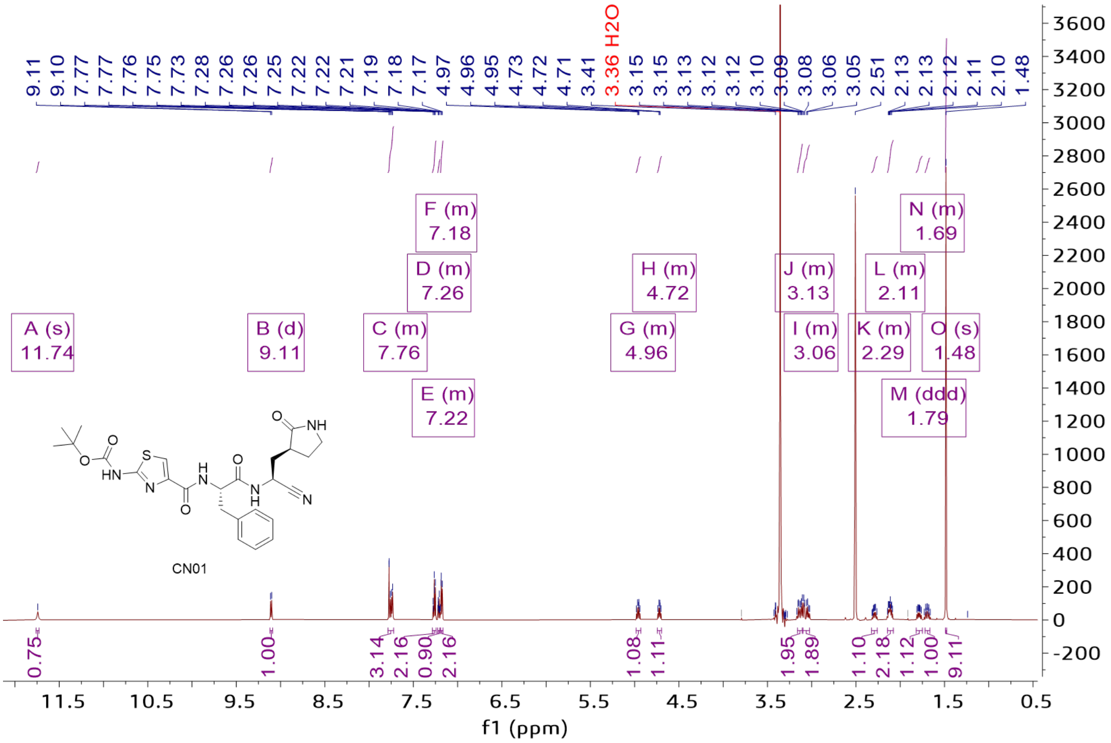
9.11 9.10 7.77 7.77 7.76 7.75 7.73 7.28 7.26 7.26 7.25 7.22 7.22 7.21 7.19 7.18 7.17 4.97 4.96 4.95 4.73 4.72 4.71 3.41 3.36 H2O 3.15 3.15 3.13 3.12 3.12 3.10 3.09 3.08 3.06 3.05 2.51 2.13 2.13 2.12 2.11 2.10 1.48
A (s) 11.74
B (d) 9.11
C (m) 7.76
D (m) 7.26
E (m) 7.22
F (m) 7.18
G (m) 4.96
H (m) 4.72
I (m) 3.06
J (m) 3.13
K (m) 2.29
L (m) 2.11
M (ddd) 1.79
N (m) 1.69
O (s) 1.48
CN01
0.75 1.00 3.14 2.16 0.90 2.16 1.08 1.11 1.95 1.89 1.10 2.18 1.12 1.00 9.11
f1 (ppm)

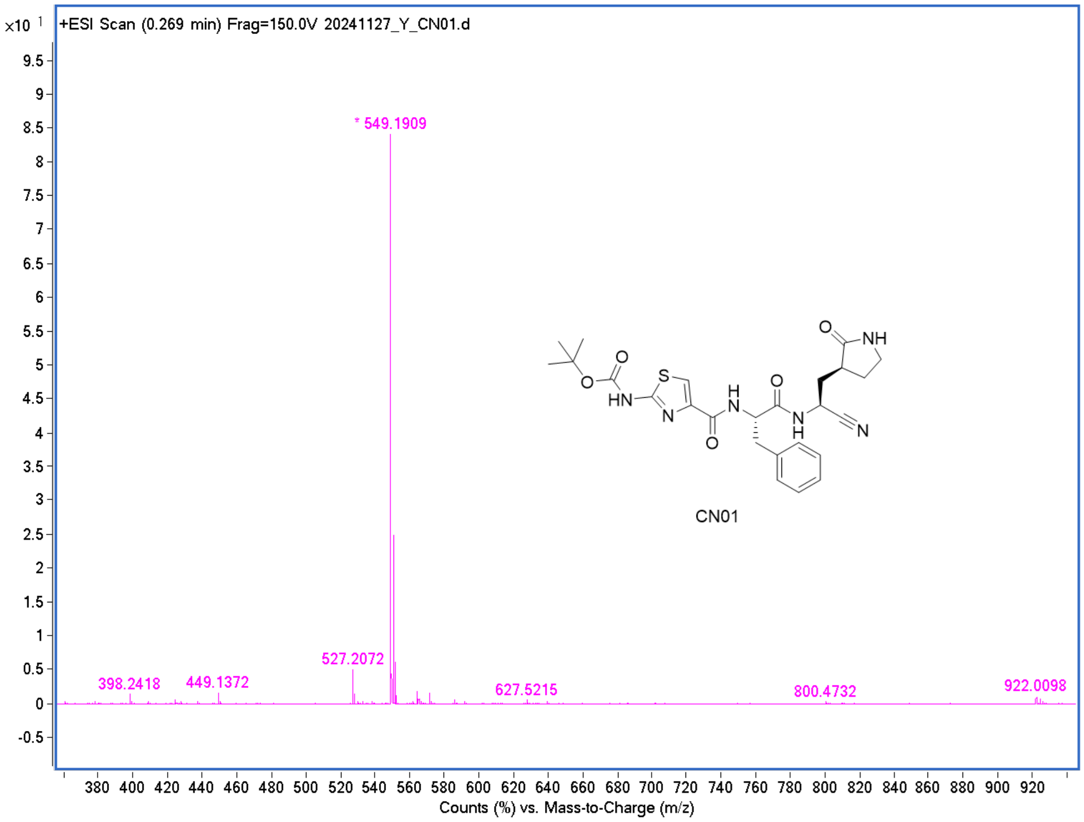
+ESI Scan (0.269 min) Frag=150.0V 20241127_Y_CN01.d
x10 1
* 549.1909
527.2072
398.2418
449.1372
627.5215
800.4732
922.0098
CN01
Counts (%) vs. Mass-to-Charge (m/z)

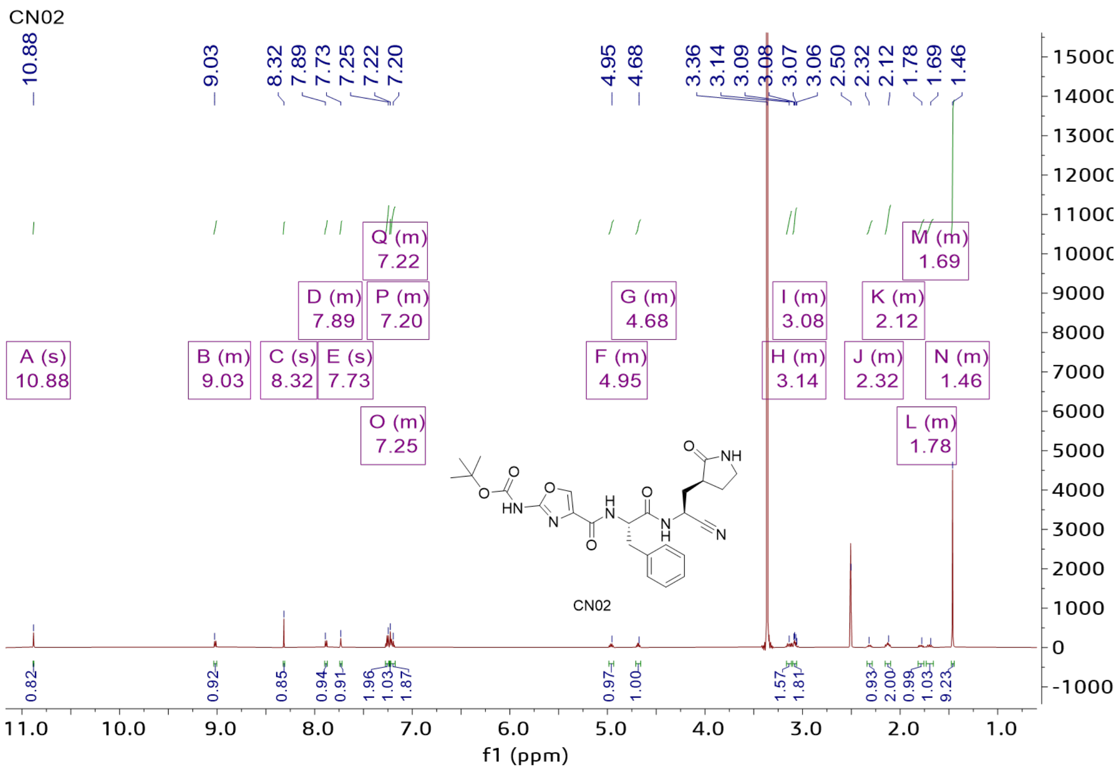
CN02
A (s) 10.88
B (m) 9.03
C (s) 8.32
D (m) 7.89
E (s) 7.73
O (m) 7.25
Q (m) 7.22
P (m) 7.20
F (m) 4.95
G (m) 4.68
H (m) 3.14
I (m) 3.08
J (m) 2.32
K (m) 2.12
L (m) 1.78
M (m) 1.69
N (m) 1.46
CN02
f1 (ppm)

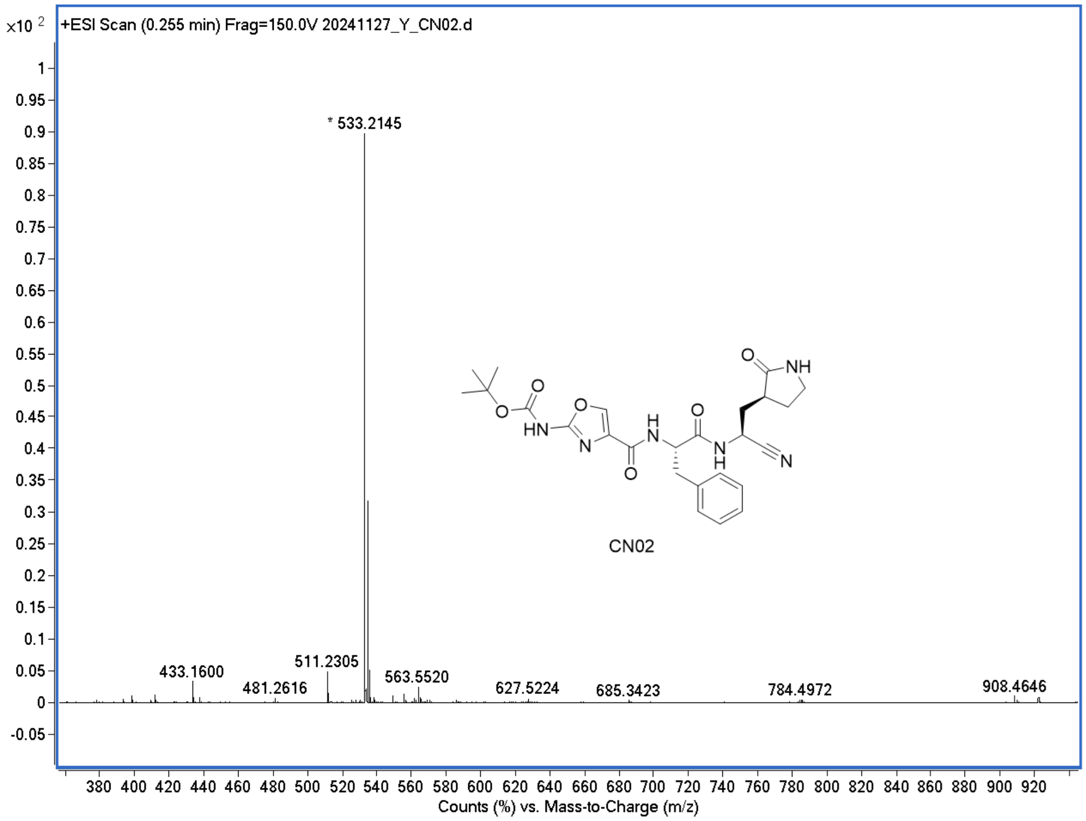
x10 2
+ESI Scan (0.255 min) Frag=150.0V 20241127_Y_CN02.d
* 533.2145
433.1600
481.2616
511.2305
563.5520
627.5224
685.3423
784.4972
908.4646
CN02
Counts (%) vs. Mass-to-Charge (m/z)

References

(1) Zhang, L.; Lin, D.; Sun, X.; Curth, U.; Drosten, C.; Sauerhering, L.; Becker, S.; Rox, K.; Hilgenfeld, R. Crystal structure of SARS-CoV-2 main protease provides a basis for design of improved alpha-ketoamide inhibitors. *Science* **2020**, *368* (6489), 409-412. DOI: 10.1126/science.abb3405.

(2) Iketani, S.; Forouhar, F.; Liu, H.; Hong, S. J.; Lin, F. Y.; Nair, M. S.; Zask, A.; Huang, Y.; Xing, L.; Stockwell, B. R.; Chavez, A.; Ho, D. D. Lead compounds for the development of SARS-CoV-2 3CL protease inhibitors. *Nat Commun* **2021**, *12* (1), 2016. DOI: 10.1038/s41467-021-22362-2.

(3) Battye, T. G.; Kontogiannis, L.; Johnson, O.; Powell, H. R.; Leslie, A. G. iMOSFLM: a new graphical interface for diffraction-image processing with MOSFLM. *Acta Crystallogr D Biol Crystallogr* **2011**, *67* (Pt 4), 271-281. DOI: 10.1107/s0907444910048675 From NLM.

(4) Evans, P. R.; Murshudov, G. N. How good are my data and what is the resolution? *Acta Crystallographica Section D* **2013**, *69* (7), 1204-1214. DOI: doi:10.1107/S0907444913000061.

(5) Potterton, L.; Agirre, J.; Ballard, C.; Cowtan, K.; Dodson, E.; Evans, P. R.; Jenkins, H. T.; Keegan, R.; Krissinel, E.; Stevenson, K.; Lebedev, A.; McNicholas, S. J.; Nicholls, R. A.; Noble, M.; Pannu, N. S.; Roth, C.; Sheldrick, G.; Skubak, P.; Turkenburg, J.; Uski, V.; von Delft, F.; Waterman, D.; Wilson, K.; Winn, M.; Wojdyr, M. CCP4i2: the new graphical user interface to the CCP4 program suite. *Acta crystallographica. Section D, Structural biology* **2018**, *74* (Pt 2), 68-84. DOI:

10.1107/s2059798317016035 From NLM.
(6) McCoy, A. J.; Grosse-Kunstleve, R. W.; Adams, P. D.; Winn, M. D.; Storoni, L. C.; Read, R. J. Phaser crystallographic software. *J Appl Crystallogr* **2007**, *40* (Pt 4), 658-674. DOI: 10.1107/S0021889807021206.
(7) Andi, B.; Kumaran, D.; Kreitler, D. F.; Soares, A. S.; Keereetaweep, J.; Jakoncic, J.; Lazo, E. O.; Shi, W.; Fuchs, M. R.; Sweet, R. M.; Shanklin, J.; Adams, P. D.; Schmidt, J. G.; Head, M. S.; McSweeney, S. Hepatitis C virus NS3/4A inhibitors and other drug-like compounds as covalent binders of SARS-CoV-2 main protease. *Scientific Reports* **2022**, *12* (1), 12197. DOI: 10.1038/s41598-022-15930-z.
(8) Emsley, P.; Cowtan, K. Coot: model-building tools for molecular graphics. *Acta Crystallogr D Biol Crystallogr* **2004**, *60* (Pt 12 Pt 1), 2126-2132. DOI: 10.1107/s0907444904019158 From NLM.
(9) Vagin, A. A.; Steiner, R. A.; Lebedev, A. A.; Potterton, L.; McNicholas, S.; Long, F.; Murshudov, G. N. REFMAC5 dictionary: organization of prior chemical knowledge and guidelines for its use. *Acta Crystallogr D Biol Crystallogr* **2004**, *60* (Pt 12 Pt 1), 2184-2195. DOI: 10.1107/s0907444904023510 From NLM.
(10) Beutner, G. L.; Young, I. S.; Davies, M. L.; Hickey, M. R.; Park, H.; Stevens, J. M.; Ye, Q. TCFH-NMI: Direct Access to N-Acyl Imidazoliums for Challenging Amide Bond Formations. *Org Lett* **2018**, *20* (14), 4218-4222. DOI: 10.1021/acs.orglett.8b01591.
(11) Dai, W.; Zhang, B.; Jiang, X. M.; Su, H.; Li, J.; Zhao, Y.; Xie, X.; Jin, Z.; Peng, J.; Liu, F.; Li, C.; Li, Y.; Bai, F.; Wang, H.; Cheng, X.; Cen, X.; Hu, S.; Yang, X.;

Wang, J.; Liu, X.; Xiao, G.; Jiang, H.; Rao, Z.; Zhang, L. K.; Xu, Y.; Yang, H.; Liu, H. Structure-based design of antiviral drug candidates targeting the SARS-CoV-2 main protease. *Science* **2020**, *368* (6497), 1331-1335. DOI: 10.1126/science.abb4489.
